# Spatio-temporal variation in pollen collected by honey bees (*Apis mellifera*) in rural-urban mosaic landscapes in Northern Europe

**DOI:** 10.1371/journal.pone.0309190

**Published:** 2025-02-04

**Authors:** Yoko L. Dupont, Thorsten J. S. Balsby, Mette B. Greve, Luna K. Marcussen, Per Kryger

**Affiliations:** 1 Department of Ecoscience, Aarhus University, Aarhus, Denmark; 2 Department of Agroecology, Aarhus University, Aarhus, Denmark; 3 Department of Agroecology, Aarhus University, Tjele, Denmark; 4 Department of Agroecology, Aarhus University, Slagelse, Denmark; Institute of Apicultural Research, CHINA

## Abstract

Pollen is a source of protein, lipids, vitamins and minerals for bees and other flower-visiting insects. The composition of macro- and micronutrients of pollen vary among different plant species. Honey bees are long-distance foragers, collecting nectar and pollen from plants within several kilometers of their hive. Availability of pollen within the foraging range of honey bees is highly dynamic, changing seasonally, and across different landscapes. In the present study, the aim was to investigate the composition of pollen collected by honey bees in rural-urban landscape mosaics typical of Northern Europe. Samples of corbiculate pollen were collected 3–9 times during the growing season by citizen scientist bee keepers from a total of 25 observation apiaries across Denmark in 2014–2015. Palynological analysis was conducted identifying 500 pollen grains per sample to pollen type (mostly plant genus). Pollen diversity denoted the number of different pollen types in a sample, while relative abundance was calculated as the proportional representation of a pollen type, if found in >1% of the sample. The quantity of pollen types across study years and sites was measured as the occurrence of each pollen type (number of samples with the pollen type present) and abundance (total number of pollen grains). Pollen diversity was highly variable, with effects of season, year, and area of green urban spaces. In terms of quantity, a few key pollen types occurred repeatedly and abundantly in the samples. Only 17 pollen types were present in >15 samples. These pollen types were consistent across study years and different landscapes. Pollen diversity may impact colony health, and hence foraging decisions by honey bees, especially in late summer. However, the bulk of the pollen collected by colonies came from a limited number of pollen sources, regardless of year and landscape context in the rural-urban landscape mosaics of Denmark.

## Introduction

Bees collect pollen as a source of protein. In addition, pollen contains lipids, vitamins and minerals [1]. Protein content and amino acid composition varies among plant species [2], and species specific protein/lipid ratio of pollen affects floral choices by pollinators [3]. In honey bees, pollen is essential for queen egg laying and larval nourishment [4–7]. Beyond macro and micro nutrients, pollen from different plants contains a host of secondary metabolites that are of relevance for honey bee health, either negatively since these are toxic [8] or positively due to antimicrobial activity [9, 10]. Hence, the botanical composition of pollen highly affects nutritional value.

A diet rich in pollen can ameliorate negative effects from varroa parasites in the pupal stage [11] and ample pollen supply is important for the survival of colonies infested by varroa mites [12]. Furthermore, a high pollen quality (high protein content) and polyfloral pollen diet can reduce mortality caused by viral infection [13, 14] and can help to mitigate the negative effects of pesticides [15, 16]. In healthy honey bee colonies, foraging effort for pollen collection is regulated by the ratio between pollen supply and pollen demand by the colony [17]. Healthy development of honey bee colonies therefore depends on pollen diet composition including quantity, nutritional quality, and floral diversity.

Like other bees, honey bees are central-place foragers [18]. Hence, colony development depends on forage availability in the landscape surrounding the hive [19]. Honey bees are long-distance foragers, flight ranges typically being up to several kilometers from the hive [20–23]. In North European landscapes dominated by farmland, more than 75% of foraging trips, as indicated by recruitment dances, occurred within 3 km of the hive, although flight distances and pollen forage plants varied over the season [24–26] and was affected by landscape composition, e.g. availability of oilseed rape or semi-natural areas [26].

Pollen collection by honey bees differ between rural and urban landscapes [27, 28]. Some studies point out specific habitat types as key floral resources for pollinators, e.g. non-forest woody vegetation with flowering trees [29] and gardens [30, 31]. Other studies emphasize key forage plants for honey bees across different habitats and landscapes. [e.g. 32–34]. Finally, genetic differences in the tendency of bees to collect pollen have been described and are thought to be a result of adaptation [35, 36].

Previous studies of pollen foraging by honey bees have often been limited temporally, and spatially to specific regions or landscape types. Most case studies of honey bee pollen supply have been carried out in landscapes dominated by agriculture [e.g. 26, 27, 37–39] although few studies have included predominantly urban [27, 28] or semi-natural landscapes [40, 41]. In contrast, the CSI Pollen project (**C**itizen **S**cientist **I**nvestigation of **Pollen,** hereafter *CSI Pollen*) collected pollen samples from private apiaries in a range of different landscapes [42, 43]. This study was coordinated by the honey bee research association COLOSS (Prevention of honey bee **CO**lony **LOSS**es) and involved citizen scientists across 24 European countries. The CSI Pollen project focused on pollen colour diversity as a rough estimate of plant diversity in the pollen supply, and high pollen colour diversity was particularly associated with urban habitats [43].

The current project employs the pollen collection of the CSI project. However, instead of analysing the pollen colour we used a palynological analysis, i.e. morphological determination of pollen by microscopy, to assess pollen diversity which is more accurate than the use of pollen colour as a proxy for pollen diversity [44, 45]. Palynological analysis of corbiculate pollen from returning foragers or bee bread stored in the comb indicates the taxonomic diversity of pollen collected by a honey bee colony [46–49] Counting the number of pollen grains of different pollen types may indicate the quantitative composition of pollen collected by honey bees [34, 42, 48, 50, 51], although pollen grains vary in size among plant species [49].

The current study further investigates the CSI Pollen data set from Denmark, where pollen samples have been identified by palynological analysis using light microscopy, in addition to the citizen science assessment of pollen colour diversity. This dataset hence provides a more detailed picture of the pollen supply to honey bee colonies in mixed urban-rural landscapes typical of an intensively farmed country in Northern Europe. We aimed to identify key pollen sources, i.e. pollen types that are collected in large quantities, in addition to the spatio-temporal variation in diversity of pollen collected by honey bees. Specifically, we address the following questions: (1) Does pollen diversity (number of different pollen types) in pollen collected by honey bee colonies vary seasonally and between study years, and is it affected by landscape composition? (2) Which are the key pollen types collected most frequently and abundantly by honey bees, and do key pollen types vary? Does the proportional abundance of key pollen types vary over the season, between study years and is it influenced by landscape composition?

## Materials and methods

### Study sites and periods

Beekeepers across Denmark participated in the CSI Pollen project (see further details in [42]). In Denmark, the Danish Beekeepers’ Association recruited citizen scientist beekeepers from their members for collecting pollen samples from their private apiaries. Study apiaries were separated by at least 6 km, to avoid overlapping 3 km foraging landscapes. Verbal informed consent was obtained from all citizen scientists involved in the study. Participants were informed about the project’s goals, procedures, and their right to withdraw at any time during meetings held before, during, and after the study. The study was based on voluntary participation in a citizen science project, and participants could stop at any point. They followed a written protocol and provided pollen samples voluntarily, with their data being fully anonymized to ensure privacy. Observation apiaries were not moved during the study. Beekeepers were provided with an illustrated step-by-step guide for installation of pollen traps and collection of samples of corbicular pollen. To document the seasonal turnover of forage plants, beekeepers collected samples from three colonies per apiary once every three weeks, from April to September during 2014 and 2015. Hence, all samples were collected during nine sampling periods, which were synchronized between the two study years, although the exact dates varied slightly between years due to inter-annual variation in weather (Table 1). Sample collection was coordinated by the Danish Beekeepers’ Association in Denmark.

**Table 1:** Periods of sampling and number of samples for each period (blue = spring, orange = early summer, green = late summer) and year (2014 and 2015).

	2014		2015	
Period	Dates	Sites (samples)	Dates	Sites (samples)
**Period1 (Apr1)**	3–7 Apr	8	4–6 Apr	15
**Period2 (Apr2)**	24–29 Apr	18	24–26 Apr	14
**Period3 (May)**	15–26 May	19	15–20 May	17
**Period4 (Jun1**	5–12 Jun	21	4–7 Jun	17
**Period5 (Jun2)**	26 Jun-1 Jul	17	26–30 Jun	18
**Period6 (Jul)**	16–30 Jul	19	16–19 Jul	16
**Period7 (Aug1)**	7–14 Aug	18	6–9 Aug	16
**Period8 (Aug2)**	28 Aug-2 Sep	17	20–30 Aug	18
**Period9 (Sep)**	18–28 Sep	16	18–20 Sep^a^	14

^a^ In 2014, a single sample was collected on September 6^th^, but included in period 9 for data analysis.

In 2014, temperatures were generally higher (average 10.0°C), and the number of sunshine hours was higher compared to the decadal average (8.8°C, 2001–2010) [52]. Precipitation was below average in spring and summer, although August was colder and wetter, compared to the decadal average [52]. In 2015, the average temperature was lower (9.5°C) than in 2014, precipitation was higher and number of sunshine hours lower than the decadal average [53]. Spring (Mar/Apr/May) was colder and had fewer sunshine hours compared to 2014 [53].

### Pollen sample collection and analysis

In each study apiary, beekeepers fitted external pollen traps to the entrances of three honey bee (*Apis mellifera*) colonies. To allow the bees to adapt to the trap, these were installed some days prior to the first pollen collection and left on the hive during the season. Pollen was collected only when traps were active, i.e. a grid was set in place at the hive entrance, forcing bees to enter the hive through grid cells, which removed pollen loads from the corbicula of foragers returning to the hive. This type of pollen trap typically removes around 10% of the pollen loads from returning foragers [54]. On each sampling occasion, pollen traps were active mostly for one day, although in some cases (23 of 153 samples in 2014; 43 of 147 samples in 2015), pollen collection was extended (up to 5 days in 2014; up to 7 days in 2015) due to adverse weather conditions, as this limits foraging activity of the bees [46]. On each sampling occasion (period), pollen loads harvested from each colony were mixed well and a random subset of 20 g (in a few cases less) of pollen were collected. Each sample for palynological analysis consisted of pollen collected from different colonies, which were pooled and mixed within each study apiary in each sampling period. In most study apiaries, pollen was collected from three colonies, although at one site in 2014, pollen was only obtained from one colony during the two collections in September. Samples were labelled and stored in the freezer (-18°C).

Palynological analysis was carried out by Lower Saxony State Office for Consumer Protection and Food Safety, Institute of Bee Science (Celle, Germany) following DIN-norm 10760 [55, 56]. This method follows a harmonized and validated procedure, which was developed for determination of botanical composition of pollen in honey (mellisopalynology), but here adapted for palynological analysis of pollen samples. In short, a 10 g random sub-sample of defrosted pollen per sample was homogenized in distilled water, the amount of water added depended on the dryness of the sample. The suspension was applied on a microscope slide using a Pasteur pipette, dried on a heat plate, and mounted with glycerine jelly. A sub-sample of 500 pollen grains was identified by light microscopy (400-1000x magnification) using a reference pollen collection [56, 57]. To ensure a homogenous examination of each slide, pollen grains were counted along lines distributed evenly across the cover slip. Only pollen types found in >1% of the sub-sample were quantified. Determination of rare pollen types (<1%) is difficult, regardless of using morphological determination [46–48] or methods based on metabarcoding [58, 59]. In the current study, we focus on plants that contribute significantly to the pollen collected by honey bees. Determination of 500 pollen grains has been shown to be sufficient for detecting both predominant and minor pollen types in a sample [60]. Pollen was identified mostly to plant genus level, more rarely to species or family level. For each sample, *pollen diversity* denotes the total number of different pollen types detected in the sample. Furthermore, for each sample, we calculated presence/absence and *relative abundance* (number of pollen grains relative to the total number of pollen grains in the sample) of the most frequently occurring pollen types (found in ≥15 samples, excluding samples with only traces of the pollen type).

To assess year-to-year variation, quantity of pollen types across all samples were assessed according to two criteria for each study year, as in Brodschneider [42]: (1) the number of samples (all study sites and dates) containing the pollen type (hereafter *occurrence*), and (2) the total number of pollen grains belonging to the pollen type in all samples (hereafter *abundance*).

### Landscape analysis

The landscape surrounding an observation apiary was defined as a circular area with a three km radius, centered on the apiary. In each study landscape and year, we carried out two different analyses: Firstly, we assessed the overall composition of each landscape by assessing the proportional representation of six basic categories. In this analysis, we aggregated land use/land cover categories in a GIS raster file referenced in GIS basemap [61]. This raster file was originally generated by gathering different authoritative datasets e.g. topographic maps, land parcel identification system data, management plans on forests, protected habitat types and cadastral maps [61]. These land use classes were further aggregated to obtain six main categories of areas using data from 2016: **Urban,** including grey urban (other building, high built up, city centre, building, industry) and green urban (low built up, gardens, and recreation area); **Agriculture** (intensive temporary and permanent crops, extensive, and non-classified agriculture); **Nature** (wet and dry natural areas); **Forest**; **Water** (lake, stream, sea); **Other** (roads, railroads, resource extraction areas) [61].

Secondly, potentially pollen providing areas were quantified within a three km landscape buffer surrounding each study apiary. Land cover/land use types in GIS, which may contain floral resources for bees were identified. These included land use types, where the typical plant community contained pollen types, which are commonly collected by honey bees, as recorded in the current study (S1 Table). The selection procedure of pollen providing land cover/land use types was inclusive, i.e. if in doubt about whether a land use type included pollen resources, it was included in the list. Based on the list of pollen providing land use types, some land use types were aggregated, resulting in the following landscape variables, which were quantified for each study landscape:

#### Area (ha) covered by flowering crops

From a national list of crop codes in Denmark [62], all known and suspected pollen providing crops were included (S1 Table). Potential pollen providing areas encompassed insect-pollinated arable crops (oilseed rape, field beans, clovers, and other seed crops), orchards (fruit trees and berry shrubs) and intensively managed grasslands with varying proportions of clover (*Trifolium* spp.) and lucerne/alfalfa (*Medicago sativa*). Crops were categorized into three levels of forage quality for honey bees (low, medium, high), using the score system of Kirk and Howe (2012) [63]. In this system, the score reflects the value of a plant as a pollen source for a given bee functional group, based on existing studies on flower-visitation rate and expert assessments. Clovers for seed production were considered high value pollen crops, while all grassland areas with clover was placed in the medium value category, as clovers in grasslands were expected to flower less intensively. In a few land use types, the major pollen providing plants of the area type was unknown, in this case the medium category was used (e.g. potted plants). Only areas of flowering crops of high and medium pollen value were included as variables in the analysis (variables *Area_Crop_high* and *Area_Crop_medium*).

#### Area (ha) of organic fields (*Area_Organic*)

The total area of organic fields was included in the analysis, as organic fields may provide a source of pollen from flowering weeds. This area included all organic fields, regardless of the value of the crop as a pollen source [64].

#### Area (ha) of high nature value

The area classified as high nature value (HNV ≥ 5), including heathlands and natural meadows was included in the analysis *(Area_HNV)*. The high nature value (HNV) indicator is based on occurrence of rare species and habitats supporting biodiversity [65, 66].

#### Area (ha) of gardens and green areas in urban areas

The area of green urban, which covered low built-up and recreational areas, was included as a variable (*Area_Green_urban*). Low built-up was predominantly gardens, while recreational areas encompassed parks, sports ground, allotment gardens, and golf courses. The area of grey urban, i.e. high built-up and other buildings, city centre and industrial areas, was not considered.

#### Area (ha) of deciduous forest

While forest themes encompass production forest and natural forest, coniferous production forests usually do not contain plant species providing pollen for honey bees [67]. In contrast, broadleaved trees, understory shrubs and herbs in natural deciduous forests include important forage plants of honey bees [67, 68]. Therefore, the area of deciduous forest was included as a variable (*Area_Deciduous_forest*).

#### Length of non-forest woody vegetation (NFWV)

A recent review suggested that lines and groups of trees and shrubs may be important landscape elements in agricultural landscapes to establish a pollinator-friendly habitat network, including important forage plants [29]. Hence, the length of hedgerows and forest edges was included as a variable *(Length_NFWV*).

#### Length of roads and railroads

Infrastructure habitats, including road verges and railroads often support a diverse flora of forage plants for bees [69]. Therefore, the total length of roads (>6m and 3-6m wide), expressways, motorways and railroads was included as a variable (hereafter *Length_Infrastructure*). Roads and railroads within 25m of areas high and low built up and industry exceeding 10 ha, were not included.

#### Distance (m) to oilseed rape field

Oilseed rape (OSR) is an important resource for honey bees and common in agricultural landscapes across Denmark. Therefore, distance from the observation apiary to the nearest OSR field was included as a variable (*Distance_OSR*).

### Data analysis

We tested the relationship between *Pollen diversity* (number of pollen types per sample) and temporal and landscape variables using a generalized linear mixed model with a Poisson distribution. Since *Area_Deciduous_forest* and *Area_HNV* showed a strong positive correlation, we tested two separate models, to avoid collinearity of landscape variables, which might affect significances and cause unreliable parameter estimates of the model [70]. The basic model was:

*Pollen diversity* = *Year* + *Period* + *Distance_OSR* + *Area_organic_field*+ *Area_Crop_medium* + *Area_Crop_high* + *Area_Green_urban* + *Length_Infrastructure* + *Length_NFWV* + Period*Distance_OSR + Period*Area_Green_urban +Period*Length_Infrastructure

In addition, model 1 included *Area_HNV* and Period*Area_HNV and model 2 included *Area_Deciduous_forest* and Period*Area_Deciduous_forest. *Period* and *Year* were discrete variables accounting for seasonal and year-to-year differences. We included *Site* as a random factor to account for the hierarchical structure of the data collection. We transformed all landscape variables using log (x+1). To facilitate comparisons of the variables effect on pollen diversity, we standardized all continuous variables.

Many of the pollen types were found only in one sample or a few of the sites. We therefore only examined the spatio-temporal patterns of the relative abundance of pollen grains which were present in at least 15 samples. This resulted in 17 pollen types that were analysed. We classified relative abundance of these pollen types based on presence/absence, as only relatively few presences existed, compared to absences. Separate analyses were carried out for each pollen type.

To test for the effect of seasonal progression on presence /absence of pollen types, we merged the nine periods into three season groups (spring/early summer/late summer, hereafter *Season*) (Table 4) to enable model convergence for most pollen types. The three seasonal groups covered spring (period 1–3), early summer (period 4–6) and late summer (period 7–9) (Table 1). We also included *Year* and *Site* as fixed effects to control for year-to-year variation and local effects. Only sites where the 17 pollen types had been detected were included in the analysis. The model thus became:

*Presence/absence of pollen type* = *Season Site Year*

We used a generalized linear model with a binomial distribution to analyse this model.

To test the effect of the landscape parameters on pollen diversity and presence/absence of pollen types, we conducted analyses for each of the landscape parameters for each of the 17 pollen types. In this analysis, we only included *Distance_OSR*, *Area_green*_*urban*, *Area_HNV*, and *Area_pollencrop_medium*, which the qualitative analysis indicated were important for pollen diversity. For the analyses, we divided the abundance of pollen grains per sample into four groups: (1) proportion of the sample ≥ 0.9, (2) 0.9 > proportion of sample ≥ 0.5, (3) 0.5 > proportion of sample > 0.05, (4) proportion of sample = 0. The abundance of pollen grains thus follows a multinomial distribution. We used a random intercept model with *Site*(*Year*) as the repeated subject. Only nine of the 17 of the most frequently occurring pollen types converged for each of the landscape variables, and five species could not converge for any of the landscape variables. For the relationship between relative abundance of pollen grains and landscape parameters, we only interpreted the positive relationships, since absence of a landscape parameter as indicated by negative associations has no information value.

We used SAS vers. 9.4 (SASInstitute, Cary, NC) to conduct the statistical analyses using proc genmod or proc glimmix.

## Results

### Study landscapes and samples

**Fig 1:**
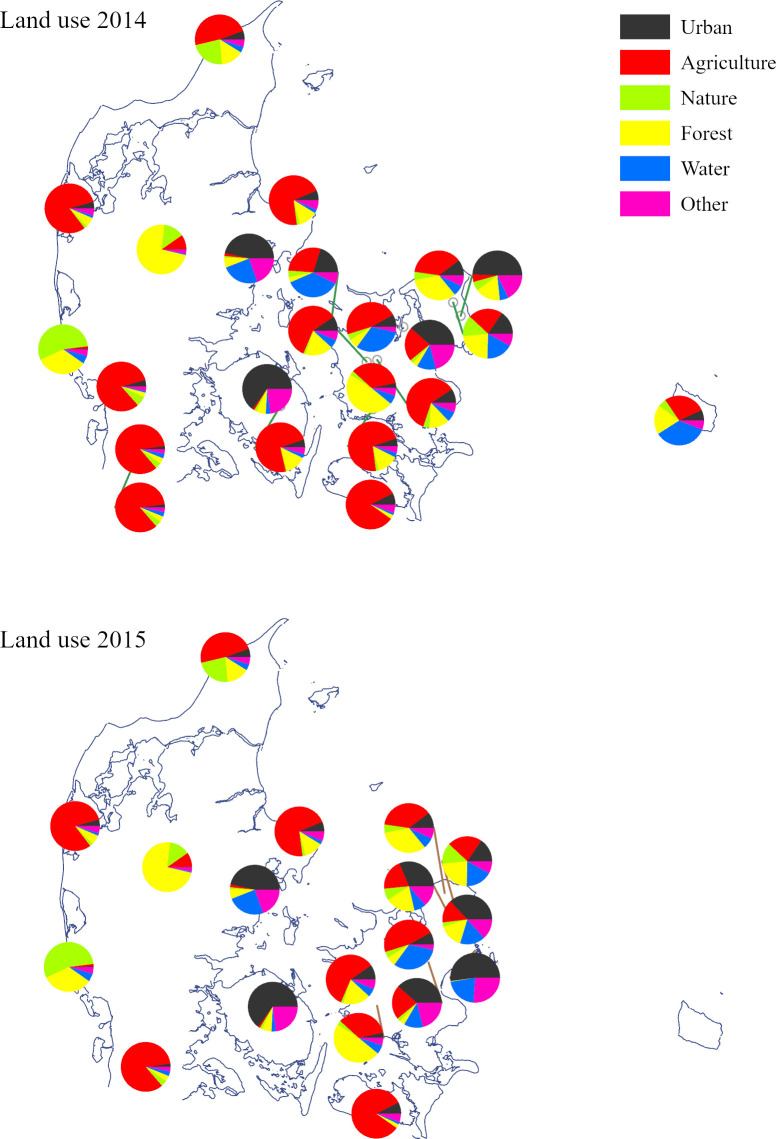
Study apiaries. Pollen samples were collected from observation apiaries located across Denmark, in 2014 (upper panel) and 2015 (lower panel). Pie charts depict the distribution of major land use types within a three km circle surrounding the apiary.

In 2014, a total of 161 samples were collected from 22 apiaries. In 2015, a total of 147 samples were collected from 18 apiaries. Of these, 15 apiaries were identical to the ones used in 2014. The landscapes surrounding the observation apiaries were mosaics of agricultural, urban, and natural areas within three kilometres of the apiary. Many of the study landscapes were dominated by agriculture, although one site did not have any agricultural areas within the three km buffer (Fig 1). The agricultural area within 3 km of the observation apiaries was on average ± SE 45.0 ± 6.3% (range 1.3–84.2%) in 2014; and average ± SE 35.5 ± 7.0% (range 0–84.3%) in 2015. The urban area was on average ± SE 14.2 ± 3.7% (range 0.4–65.0%) in 2014, and average ± SE 19.1 ± 4.8% (range 0.4–65.0%) in 2015. The area of open nature was on average ± SE 7.1 ± 2.4% (range 0.1–55.0%) in 2014 and average ± SE 7.8 ± 3.1% (range 0–55.0%).

Pollen samples were collected from a total of 16–21 apiaries per period in 2014 and 15–18 apiaries per period in 2015, except for period 1 in 2014, during which only eight apiaries were included. The total number of samples collected varied among observation apiaries and years, as some beekeepers did not succeed in taking samples during all nine sampling periods each year. In 2014, the number of samples collected per apiary was on an average ± SE of 6.95 ± 0.56 (range 3–9). In 2015, an average ± SE of 8.17 ± 0.09 (range 5–9) samples were collected per apiary.

### Overall number of pollen types and plant families

Many pollen types were detected in the pollen samples (Table 2, S2 Table), although many pollen types were rare, and only 17 pollen types were found in 15 samples, when traces of pollen (< 1% of a sample) were not considered. Most pollen types occurred in both study years. However, 19 pollen types in 2014 and 35 pollen types in 2015 were unique to one study year.

**Table 2:** Number of sites, samples and pollen types in 2014 and 2015.

	Sites	Samples	Pollen types^a^	Plant families^b^
**Samples from 2014**	22	153	176	76
**Samples from 2015**	18	147	192	78
**Found in both 2014 and 2015**	15	-	157	67
**Overall (2014–2015)**	25	300	211	85

^a^ Total number of different pollen types detected across all samples.

^b^ Total number of plant families represented among pollen types detected across all samples.

The most diverse plant families were Asteraceae (19 pollen types), Fabaceae (15), Rosaceae (15) and Apiaceae (10) (S2 Table). Many plant families (46 out of 85) were represented by only one pollen type in the data set.

### Spatio-temporal variation in pollen diversity

**Fig 2:**
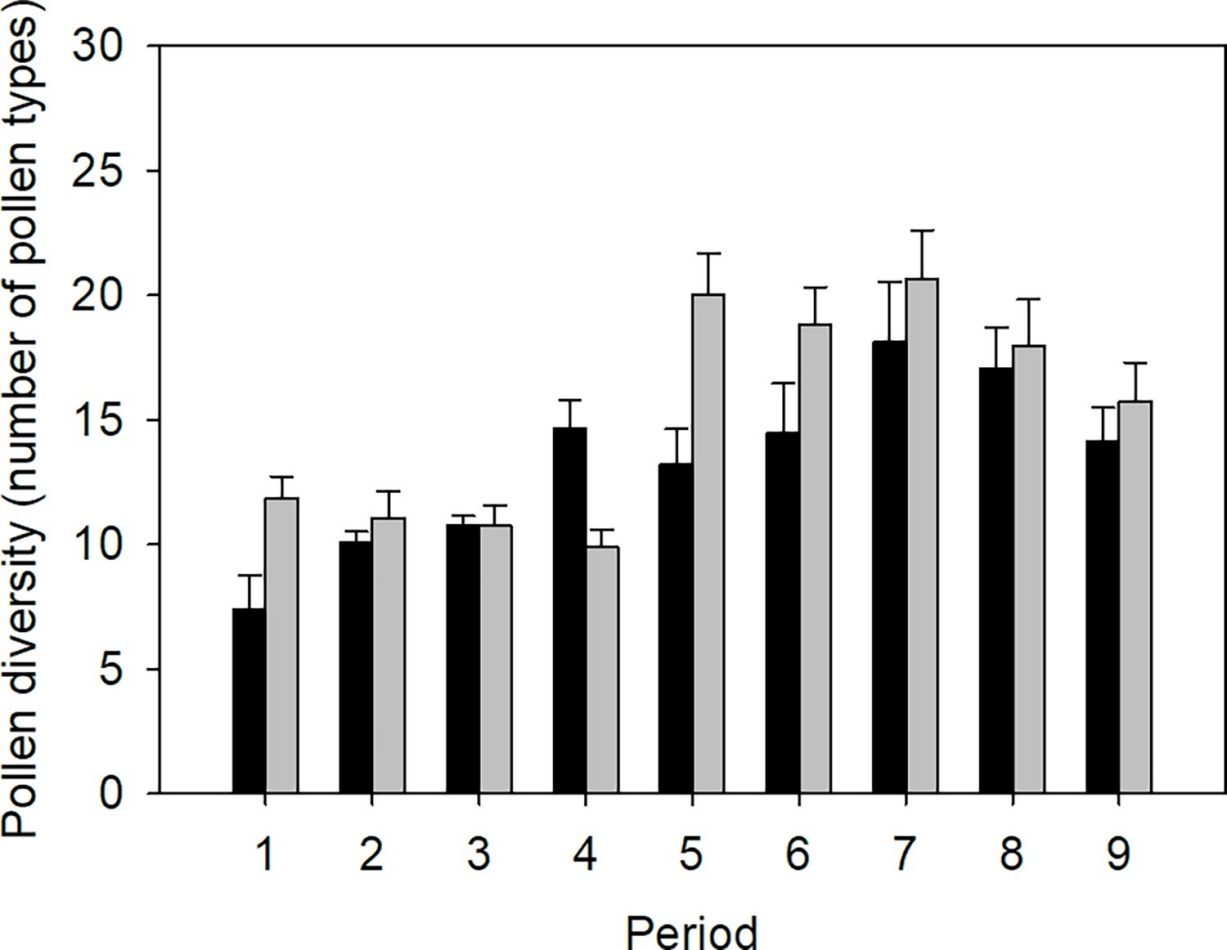
Seasonal variation in pollen diversity. Total number of different pollen types per sample in each period (average ± SE) in 2014 (black columns) and 2015 (grey columns). Period 1 is early April, period 2 late April, period 3 mid-May, period 4 early June, period 5 late June, period 6 mid-July, period 7 early August, period 8 late August, and period 9 mid to late September. For sampling dates, see Table 1.

Based on original data, pollen diversity was on average ± SE 13.69 ± 0.5 (range 2–41) pollen types per sample in 2014, and 15.31 ± 0.6 (range 4–38) pollen types per sample in 2015. However, pollen diversity varied seasonally. In general, pollen samples in spring (April-May) contained fewer pollen types than samples from early and late summer (June-September). Pollen diversity peaked in early August in both 2014 and 2015 (Fig 2).

### Pollen diversity

Overall, the significance and estimates from the two GLMM models relating pollen diversity per sample to time and landscape variables were similar for both models. We have therefore not distinguished between the two models. *Pollen diversity* per sample was significantly higher in 2015 compared to 2014 (Table 3). *Period* also caused significant variation in *Pollen diversity* (Table 3) with a higher diversity in late season (periods 5–9) compared to early season (periods 1–4). Pairwise comparisons showed that most periods differed significantly (S3.1 Table in S1 Appendix).

**Table 3:** GLMM on pollen diversity for model 1 and 2 including estimates (Est.) for the effect of the continuous variables. Statistically significant p-values are in bold.

Variable	Model 1				Model 2			
	df	F	p	Est.	df	F	p	Est.
***Period***	8. 235	23.22	**< .0001**		8. 235	23.11	**< .0001**	
***Year***	1. 235	7.12	**0.008**		1. 235	5.39	**0.011**	
***Distance_OSR***	1. 235	1.86	0.174	0.039	1. 235	1.37	0.243	0.036
***Area_ organic fields***	1. 235	1.41	0.236	-0.041		1.05	0.307	-0.034
***Area_Crop_medium***	1. 235	0.04	0.851	-0.006	1. 235	0.08	0.78	-0.008
***Area_Crop_high***	1. 235	0.42	0.516	0.023	1. 235	0.73	0.394	0.031
***Area_Green_urban***	1. 235	19.38	**< .0001**	0.184	1. 235	20.92	**< .0001**	0.185
***Length of infrastructure***	1. 235	0.23	0.632	-0.027	1. 235	0.37	0.543	-0.034
***Length_NFWV***	1. 235	3.44	0.065	-0.049	1. 235	3.64	0.058	-0.050
***Area_HNV***					1. 235	1.10	0.295	-0.029
***Area_Deciduous_forest***	1. 235	0.33	0.569	-0.001				
Area_Green_urban*Period	8. 235	4.62	**< .0001**		8. 235	4.49	**< .0001**	
Length_railroad*Period	8. 235	0.60	0.775		8. 235	0.45	0.893	
Area_Deciduous_forest*Period	8. 235	1.04	0.404					
Area_HNV*Period					8. 235	1.95	0.054	

The landscape variable *Area_Green_urban* showed a significant positive relationship with *Pollen diversity* (Table 3). The effect of the green urban areas differed across the season, as indicated by the significant interaction effect Area_Green_urban*Period (Table 3). The other main factors and the interaction effects did not show significant effects on *Pollen diversity* (Table 3).

### Year-to-year variation in occurrence and abundance of pollen types in honey bee corbiculate pollen

**Fig 3:**
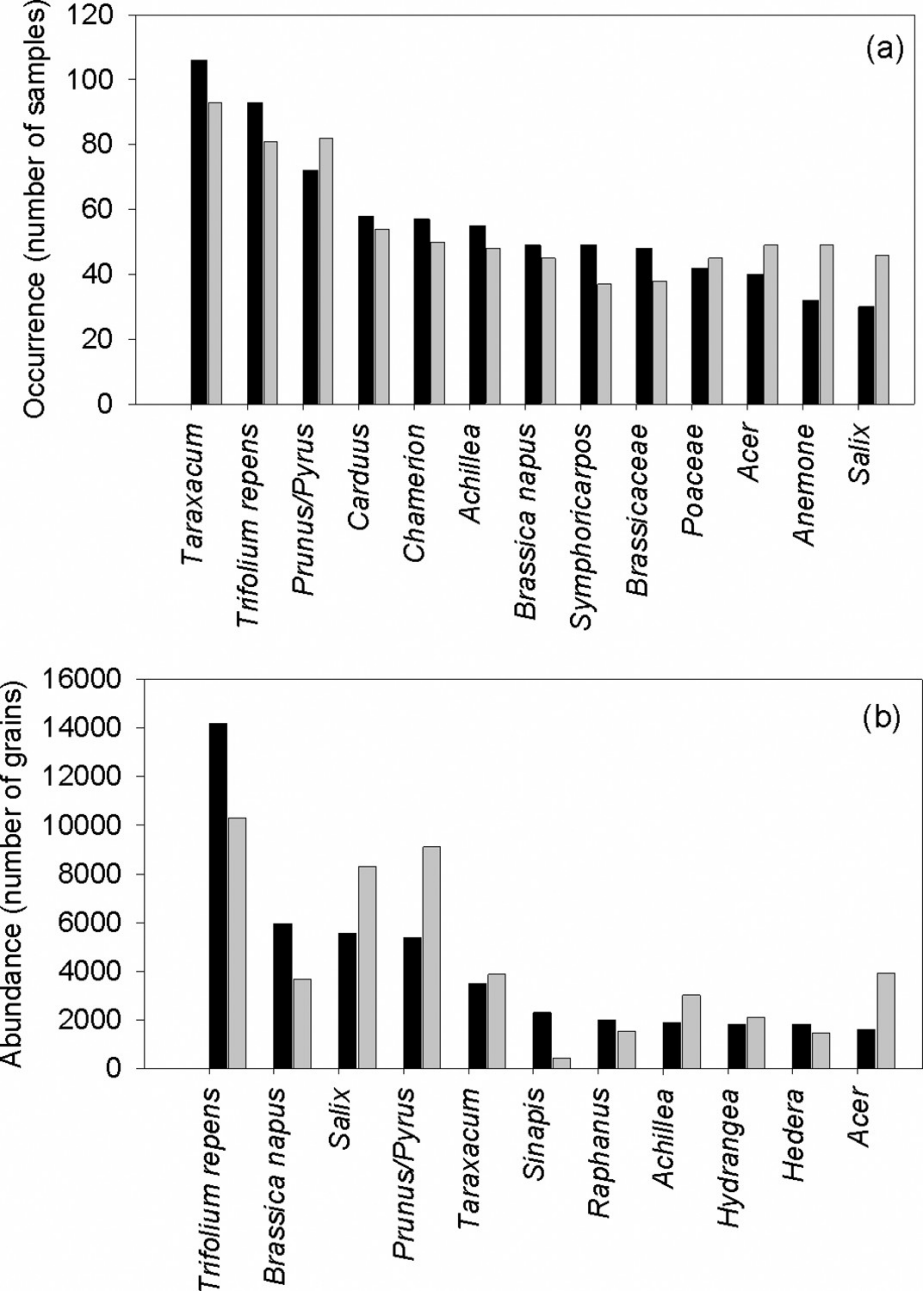
(a) Occurrence (number of samples) of the 10 most frequently occurring pollen types in samples collected in 2014 (black columns) and 2015 (grey columns). *Symphoricarpus* and Brassicaceae were only among the 10 most commonly occurring pollen types in 2014, while *Acer*, *Anemone* and *Salix* were only among the 10 most commonly occurring pollen types in 2015. (b) Abundance (total number of pollen grains) of the 10 most abundant pollen types in samples collected in 2014 (black columns) and 2015 (grey columns). *Sinapis* was only among the 10 most abundant pollen types in 2014, while *Acer* was only among the 10 most abundant pollen types in 2015.

Based on original data, the occurrence of pollen types was highly skewed, with each pollen type occurring on average ± SE of 11.97 ± 1.3 (range 1–106) samples in 2014, and 11.85 ± 1.3 (range 1–93) samples in 2015. Looking at the whole data set, including all samples across all study sites and periods within each study year, some general patterns of bee pollen collection emerge. A limited number of plants occurred frequently and in high abundance in the pollen samples. The most frequently occurring pollen types in both years were *Taraxacum* (106 samples in 2014 vs 93 samples in 2015), *Prunus/Pyrus* (102 vs 117 samples), *Trifolium repens* (93 vs 81 samples), *Carduus* (58 vs 54 samples) and *Chamerion* (57 vs 50 samples). In 2014, these were followed by *Achillea* (55 samples), *Symphoricarpos* (49 samples) and *Brassica napus* (49 samples), while in 2015, other common pollen types were *Anemone* (49 samples), *Acer* (49 samples) and *Achillea* (48 samples) (Fig 3A). Some of these pollen types generally had a low abundance in the samples, often present only as traces (< 1%), e.g. *Chamerion* and *Symphoricarpos*. Hence, this list deviates from the 17 most frequently occurring pollen types included in the statistical analysis.

When considering the total abundance of pollen types, *Trifolium repens* was by far the most abundant pollen type in both 2014 (14190 pollen grains) and 2015 (10310 pollen grains). Other abundant pollen sources in 2014 were *Brassica napus* (5960), *Salix* (5570), *Prunus/Pyrus* (5400), *Taraxacum* (3510), *Sinapis* (2305), *Raphanus* (1995) and *Achillea* (1880). In 2015, *Prunus/Pyrus* (9120) and *Salix* (8320), followed by *Acer* (3940), *Taraxacum* (3870), *Brassica napus* (3675), *Achillea* (3015) and *Hydrangea* (2120) (Fig 3B). For further information on occurrence and abundance of pollen types in 2014 and 2015, see S2 Table.

### Seasonal patterns of occurrence and abundance of pollen types in honey bee corbiculate pollen

In both study years, pollen of *Salix* was highly abundant in early spring (period 1 and 2) and commonly occurred in the pollen samples (data on occurrence and abundance of pollen types in periods in 2014 and 2015, see S2 Table). In addition, samples from early April (period 1) contained to a lesser extent pollen from early spring flowers, in particular *Scilla* and Ranunculaceae (possibly *Erantis*), and in 2015 also Hyacinthaceae. From late April to May (period 2–3 in 2014) or early June (period 4 in 2015), samples often contained *Prunus/Pyrus* and *Brassica napus*, often in large proportions. Pollen of *Prunus/Pyrus* was found almost throughout the season, although the amount dropped considerably from early (period 4 in 2014) or late (period 5 in 2015) June. In both years, pollen of *Acer* and *Aesculus* were commonly and abundantly collected by the bees during a short period in mid May to early June (period 3–4). In contrast, *Taraxacum* often occurred in the pollen samples from late April until September (periods 2–9), but were rarely abundant, although abundance increased towards late summer.

In early-mid summer (June-July, periods 4–6), bees collected pollen from a wider range of plant species compared to the spring period (periods 1–3). In early summer (early June, period 4), *Hydrangea*, *Rubus* (mostly 2014), *Sinapis* (2014)/*Raphanus* (2015) and *Vicia faba* were often and/or abundantly collected, while in late summer (July-early August, periods 6–7), *Trifolium pratense*, *Buddleja*, *Eupatorium* (2014 only) and *Sorbus* (2015 only) were found in several samples, sometimes in high numbers. However, the most frequently occurring and abundant pollen type throughout the summer (June-September, periods 4–9) was *Trifolium repens*. A few pollen types were often detected during mid-late summer (July-August, periods 6–8), although rarely abundant in the samples. These included *Achillea*, *Carduus*, *Chamerion* and *Symphoricarpos* in both study years, in addition to *Lavandula*, *Tilia*, *Ligustrum*, *Melilotus* and *Viola*, which often occurred in low abundances in samples from July 2015 (period 6). Furthermore, pollen grains of Poaceae occurred as traces in many pollen samples from June-early August (periods 4–7).

In late summer/early autumn (periods 8–9), nutrient catch crops such as *Sinapis* and *Raphanus* often occurred in the samples at high abundance, in addition to ornamental plants such as *Aster/Solidago*, *Sedum* (2014)/Crassulaceae (2015) and *Hedera*.

### Year, site, season and landscape effects on abundance of most commonly occurring pollen types per sample

The likelihood of encountering specific pollen types did not differ significantly between 2014 and 2015 for any of the nine species where models could converge (Table 4). The presence of pollen grains showed no significant difference between sites for any of the 14 pollen species where models could converge (Table 4).

**Table 4:** Test for the effect of site, period (season) and year on abundance (likelihood of presence). Statistically significant p-values are in bold.

Pollen type analysis	Site			Season			Year			Model^a^
	df	Χ^2^	p	df	Χ^2^	p	df	Χ^2^	p	
***Acer***	28	9.08	1.000	2	63.60	**1.55E-14**	1	2.58	0.108	Full
***Achillea***	15	17.58	0.285	2	54.00	**1.88E-12**	1	0.00	1.000	Full
***Aesculus***	13	2.20	1.000	2	38.43	**4.52E-09**				Full
***Aster/Solidago***	9	5.14	0.822	2	27.82	**9.11E-07**				Full
***Brassica napus***	28	14.66	0.982	2	74.24	**7.58E-17**	1	0.82	0.367	Full
***Carduus***	10	4.86	0.901	2	20.14	**4.23E-05**	1	0.47	0.493	Full
***Hedera***				2	45.47	**1.34E-10**				Period
***Papaver***	9	7.68	0.567	2	25.81	**2.48E-06**				Full
***Prunus/Pyrus***	33	31.18	0.558	2	129.99	**5.94E-29**	1	0.40	0.526	Full
***Raphanus***	8	6.94	0.543	2	20.07	**4.39E-05**				Full
***Rhus***	9	4.04	0.909	2	14.76	**6.23E-04**	1	0.02	0.887	Full
***Rosa***	12	3.98	0.984	2	32.28	**9.81E-08**				Full
***Salix***				2	164.43	**1.97E-36**				Period
***Sinapis***	10	3.82	0.955	2	16.33	**2.84E-04**	1	0.46	0.499	Full
***Taraxacum***	26	23.79	0.588	2	22.77	**1.14E-05**	1	0.01	0.915	Full
***Trifolium pratense/incarnatum***	11	5.84	0.884	2	28.01	**8.29E-07**	1	0.52	0.469	Full
***Trifolium repens***				2	120.31	**7.50E-27**				Period

^a^For the species where the full model could not converge, we used a simpler model to test the effect of period. This column “model” indicates which model had been used.

The three seasonal groups (spring/early summer/late summer) showed significant difference for all pollen types tested with the full model and for the pollen species where the model only included period (Table 4). The seasonal pattern differed between some species. The presence of *Salix* peaked in spring (period1-3). *Brassica napus* and *Aesculus* both peaked in the spring and seemed almost absent in late summer. *Acer*, *Prunus/Pyrus* and *Rhus* also showed presence in the spring but were also present in early summer. *Achillea*, *Carduus*, *Trifolium pratense/incarnatum*, *Papaver*, *Raphanus*, *Rosa*, *Sinapis* and *Trifolium repens* indicated a general presence in early and late summer and absence in spring. *Hedera*, *Taraxacum* and *Aster/Solidago* mainly occurred in late summer (Table 4, S3.1 Fig in S1 Appendix).

For most species, the likelihood of encountering pollen showed no relation to the landscape parameters (S3.2 Table in S1 Appendix). The exceptions were the positive relationships between *Rhus* and *Distance_OSR*, *Aster/Solidago* and *Area_Green_urban*, and *Taraxacum* and *Area_HNV* (S3.2 Table in S1 Appendix).

## Discussion

Honey bees collected pollen from a range of different plant species. Pollen diversity per sample varied seasonally with a peak in August and was significantly higher in 2015 than 2014. In contrast, a small number of key pollen types were commonly and/or abundantly found in honey bee collected pollen across study years and landscapes. Landscape parameters had little influence on pollen collected by the bees, only the area of green urban spaces affected pollen diversity and the relative abundance of very few pollen types.

### Diversity of pollen types in honey bee corbiculate pollen

Honey bees have been shown to increase foraging distance towards late summer in a typical Northern European landscape mosaic, consisting mostly of farmland and to a lesser extent urban areas, forest and grassland [24, 25]. Floral resource scarcity has been documented in the same region during late summer [71]. Expanding foraging range may to some extent counteract dwindling in floral resources, however, it may also signal that the bees are selecting most suitable plants in supplementing their storage. In accordance with this interpretation, foraging distance was positively correlated with the sugar content of nectar collected by foragers [24]. A high pollen diversity could reflect that honey bee scouts expand their foraging range in late summer, and hence encounter more diverse sources of forage. In accordance with the studies mentioned above, pollen diversity peaked in late summer in the current study. Furthermore, pollen diversity was significantly higher in 2015 than 2014. This could be due to adverse weather conditions for foraging in 2015, which may have forced the bees to expand their foraging range to include a wider variety of plants. In contrast, during spring, flowers from mass flowering trees, shrubs and herbs flower abundantly, often within short distances of the hive. In spring, bees may use a limited number of pollen sources, consistent with the low pollen diversity observed in the current study, as well as similar studies in Austria [42] and Wales [51].

### Honey bee collected pollen: Temporal specialization on mass flowering plants

Honey bees are known as generalist foragers, visiting a wide range of plants for nectar and pollen [72, 73]. In the current study, honey bees collected pollen from a total of >100 pollen types, and the average pollen diversity of pollen samples was 14–15 pollen types. However, as in previous studies [43, 50], we also found that honey bee collected pollen was dominated by a few pollen types from plant species rich in floral resources, even when diverse pollen sources were found in the landscape surrounding the apiary. In contrast to other insect flower-visitors, visitation by honey bees is strongly influenced by local and landscape level flower availability [74, 75], and honey bees are preferentially attracted to mass-flowering plants [76].

Our study showed that honey bees temporally specialize in collecting pollen from widespread and abundantly flowering plants, and that these pollen types are constant across different landscape mosaics consisting of mixed rural and urban landscapes and across different study years. These key pollen types included mass flowering crops as well as wild plant species. In contrast to solitary insects or bumblebees which produce only small annual colonies, large floral resources are needed to support large perennial honey bee colonies. A honey bee colony consumes 17–34 kg pollen per year, depending on colony size [5, 77].

### Common and abundant pollen types

The key pollen types were consistent over the two study years, and similar to those found in a study investigating botanical composition of bee bread from apiaries across Denmark in 2020–2021 [34]. The most frequently occurring pollen types (presence in many samples) was *Taraxacum*, *Prunus/Pyrus*, *Trifolium repens*, *Carduus* and *Epilobium*. Measured as number of pollen grains, by far the most abundant pollen type in the samples was *Trifolium repens*. This plant species is widespread in Denmark, found in grass-clover leys in agricultural areas, lawns in urban areas, as well as in road verges and semi-natural grasslands [78]. It provides ample nectar [79] in addition to pollen, and flowers from mid-June until early August [80], i.e. a significant proportion of the active season of the honey bees. Other abundant pollen types in the samples were *Salix*, *Prunus/Pyrus*, *Brassica napus*, *Taraxacum*, *Acer*, *Hydrangea*, *Achillea*, *Raphanus* and *Hedera*. Abundance of the most commonly occurring pollen types was shown to be most strongly associated with the season, and rarely with landscape parameters. Therefore, a small number of forage plants with a seasonal progression of flowering appears to be of key importance in the pollen collection of honey bees across the different rural-urban mosaic landscapes of the current study.

*Salix* spp provide a key source of forage for honey bees in early spring, supporting the early season development of honey bee colonies. *Salix* consists of *c*. 20 species in Denmark [80], and although each species blooms only for a short time, segregated flowering of different individuals and species may provide floral resources for an extended period during early spring. The dominance of *Salix* pollen in early spring emphasizes the key importance of this plant genus as a pollen source at this time of the year, although pollen may to a lesser extent be collected from other early spring flowers such as *Skilla* and *Hyacinthaceae*. Building up the colony in early spring is essential for the performance of the colony during the season [12].

As spring progresses, a series of mass flowering species, including fruit trees (*Prunus/Pyrus*) and maple (*Acer*), oilseed rape (*Brassica napus*) and dandelion (*Taraxacum*) begin to bloom. Climate conditions, especially temperature cues, affect flowering phenology, including onset and length of flowering [81]. Furthermore, foraging by bees is constrained by adverse weather conditions [82, 83]. This could result in year-to-year and local variation in the proportional representation of different pollen types in bee collected pollen, especially the amount of pollen from plant species with relatively short flowering phenologies such as the key forage plants flowering in mid-late spring. Furthermore, as samples from pollen traps are snap shots of the pollen flow into the hive, the botanical composition of bee collected pollen may vary, depending on the exact timing of sampling [83].

In addition to the ubiquitously flowering *Trifolium repens*, abundant pollen types confined to early and late summer included a range of different species, the most abundant pollen types being the wild flower *Achillea*, the ornamental plant *Hydrangea*, and the catch crop *Raphanus*. In addition, the late flowering *Hedera* was a key pollen source only in late summer. During the summer period, some pollen types frequently occurred in samples, although never in high numbers. These included *Carduus*, *Epilobium* and *Symphoricarpus*. Most notably, the latter occurred in 49 samples in 2014 and 37 samples in 2015, but with < 5 pollen grains in each sample. These plants are known to produce large amounts of nectar and may be visited for nectar rather than pollen [79, 84, 85]. This may apply also to e.g. *Lavandula*, *Tilia* and *Ligustrum*. Pollen found in low abundance, but high occurrence may therefore indicate that these forage plants are visited frequently for nectar, although pollen is not collected in large amounts by the honey bees. The colony strength, i.e. number of adult bees in a colony, typically peaks in mid-summer in Denmark, therefore ample nectar is needed to support honey bee colonies at this time of the season [86].

### Effects of pollen diversity and abundance on honey bee health and survival

The amount of pollen harvested in spring affects colony reproductive performance and health throughout the season [12]. Building up provision in spring can safeguard the colony during periods of flower scarcity and/or unfavorable foraging weather [12]. Spring-flowering oilseed rape is the main source of honey in Denmark and UK [87, 88], similarly a high pollen flow is expected to support the rapid growth of colonies early in spring. But later in the season, foraging decisions by honey bees may be affected more by pollen protein quality and diversity than pollen quantity. Honey bees switch to pollen sources having higher protein content rather than abundant flowers in late summer, when availability of protein is generally low [89]. Pollen quantity and protein content appear to impact honey bee health more than pollen diversity *per se* [14, 90]. However, pollen from different plant species vary considerably in macro and micronutrient composition [3], and honey bees are more selective in their choice of pollen sources, compared to nectar plants [91]. Honey bee colonies have been documented to differentially recruit foragers to pollen sources, which balances nutritional deficiencies, when the colony is deprived of essential amino acids [92] or fatty acids [93]. Hence, pollen collection by honey bee colonies is a complex interplay of external factors such as climate and flowering phenologies, in addition to intrinsic colony-level factors, including nutritional and health status of the bees.

### Landscape and context dependency of pollen collection

Agricultural landscapes in Northern Europe are spatio-temporally variable in floral resource availability [26, 71], and range from structurally simple, resource poor landscapes to complex landscapes with semi-natural, flower-rich habitats [94, 95]. Considering this variability, pollen diversity and composition of the predominantly collected pollen types were surprisingly little affected by landscape factors in the current study. The surroundings of the apiaries had varying proportions of agriculture, urban and natural areas, and the agricultural area ranged from 0 to 84% of the landscape. Yet, few landscape parameters were associated with honey bee pollen collection. Only the area of green urban spaces was associated with pollen diversity, and with the relative abundance of the garden ornamental *Aster/Solidago*.

In accordance with Brodschneider et al. [43], a higher pollen diversity was observed in urban areas. Cities and towns, especially domestic gardens, allotments and other urban green spaces, provide important flower-rich habitats for pollinators, in patches in between impervious surfaces [96]. For pollinators such as honey bees, which have a foraging range exceeding a single green space, this urban mosaic provides a temporally stable forage supply [97]. Honey bees foraging in urban areas may therefore collect pollen from a wide range of plant species, increasing pollen diversity of their diet.

Many garden plants are used as pollen sources by honey bees, but only the abundance of *Aster/Solidago* was positively related to area of urban green spaces. Pollen collected by honey bees in farmland are typically less diverse. Hence, in rural-urban mosaic landscapes, the high diversity of forage plants in cities and towns may increase nutritional value of pollen collected by bees [32].

In conclusion, a high diversity of pollen types (>100) was collected by honey bees in mixed rural-urban landscapes across Denmark. Pollen diversity varied seasonally, peaking in late summer, and varying from year-to-year. While pollen diversity can be variable and associated with landscape composition and foraging range of the bees, only a few pollen types (< 20) were collected frequently and abundantly. Although honey bees are known as generalist pollinators, pollen collection was concentrated on mass blooming crops, ornamentals and wild plants. Honey bee pollen collection was temporally specialized with a turnover of key forage plants flowering at different times during the season. Despite the effects of landscape composition on pollen diversity, the major pollen plants were consistent across different landscape mosaics.

## Supporting information

S1 Table Potential pollen providing crops. Basemap object code and object name of GIS themes according to Levin (2019) [62]. Value as a source of pollen was assessed based on the scoring system by Kirk and Howe (2012) [63].
(XLSX)

S2 Table Occurrence and abundance of pollen types in pollen samples collected from observation apiaries in 2014 and 2015. Occurrence is the number of samples containing the pollen type. Abundance is number of pollen grains pooled across all samples. x denotes traces of the pollen type (< 5 pollen grains per sample).
(XLSX)

S1 Appendix Statistical analysis. S3.1 Fig. Least square means ± SE for the three seasonal groups for each of the pollen species. Note least square means estimates for *Salix*, *Aesculus*, *Aster Solidago* type, *Hedera*, *Rosa*, *Papaver* type, *Trifolium repens* and *Raphanus* type were all estimated by the model that only included period. S3.1 Table. Pairwise comparison. Least square means differences in pollen diversity for period in Model 1 and Model 2, respectively. df = 235. S3.2 Table. Effect of landscape variables on abundance (presence of pollen species). Note that all tests were made as univariate tests on log transformed distance and area. If the G matrix was not positive definite, we omitted the test from the table.
(DOCX)
