# Supplementary material for: Spatio-temporal variation in pollen collected by honey bees (Apis mellifera) in rural-urban mosaic landscapes in Northern Europe
Source: PLoS One. 2025 Feb 4;20(2):e0309190. doi: 10.1371/journal.pone.0309190 (PMC11793741; doi:10.1371/journal.pone.0309190)
Supplement: S1 Appendix — S3.1 Fig. Least square means ± SE for the three seasonal groups for each of the pollen species. Note least square means estimates for Salix, Aesculus, Aster Solidago type, Hedera, Rosa, Papaver type, Trifolium repens and Raphanus type were all estimated by the model that only included period. S3.1 Table. Pairwise comparison. Least square means differences in pollen diversity for period in Model 1 and Model 2, respectively. df = 235. S3.2 Table. Effect of landscape variables on abundance (presence of pollen species). Note that all tests were made as univariate tests on log transformed distance and area. If the G matrix was not positive definite, we omitted the test from the table. (DOCX) [file pone.0309190.s003.docx]

## Supporting Information

**S3 Appendix. Statistical analysis**

Figure S3.1. Least square means ± SE for the three seasonal groups for each of the pollen species. Note least square means estimates for *Salix*, *Aesculus*, *Aster Solidago* type, *Hedera*, *Rosa*, *Papaver* type, *Trifolium repens* and *Raphanus* type were all estimated by the model that only included period.


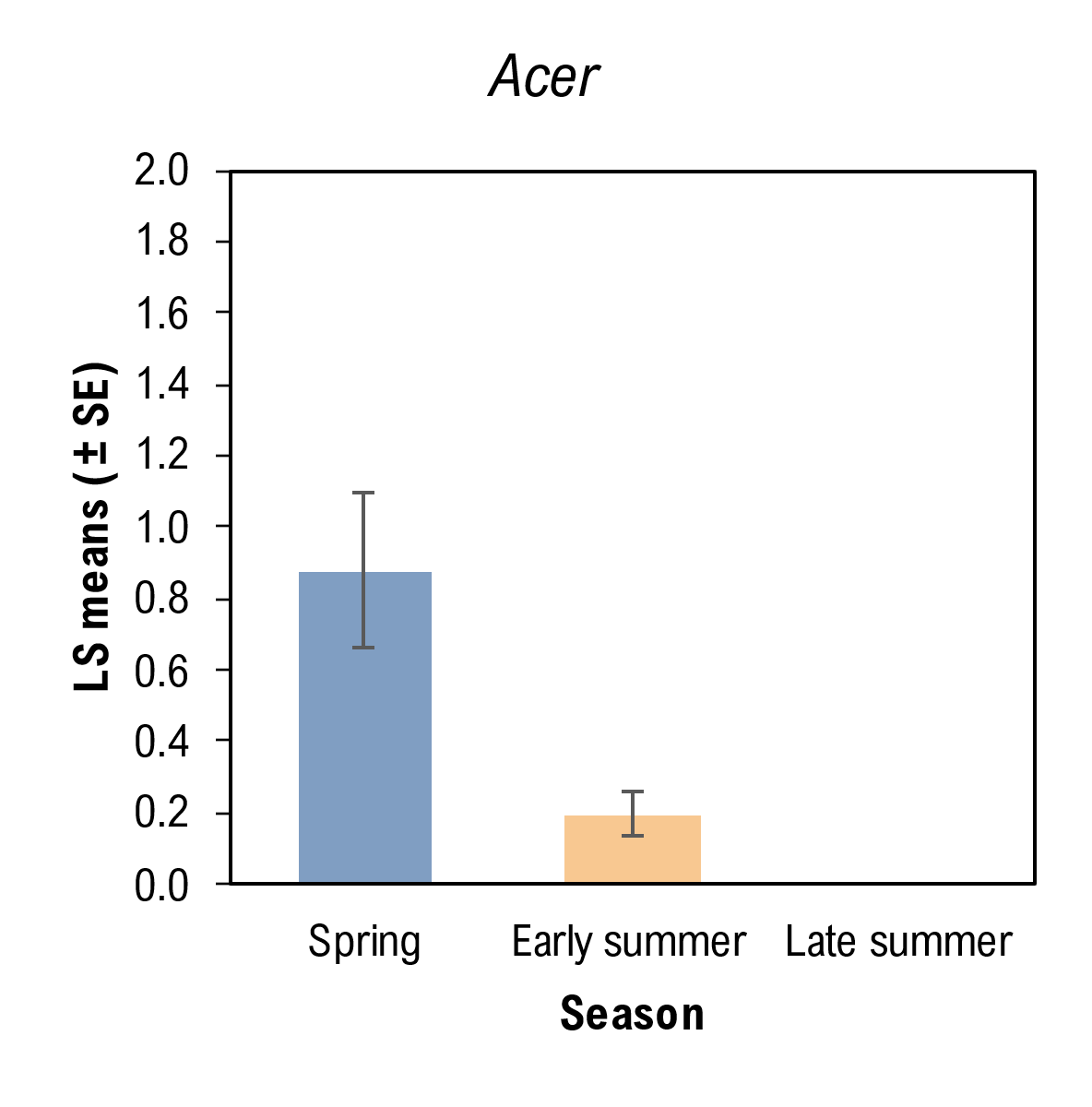

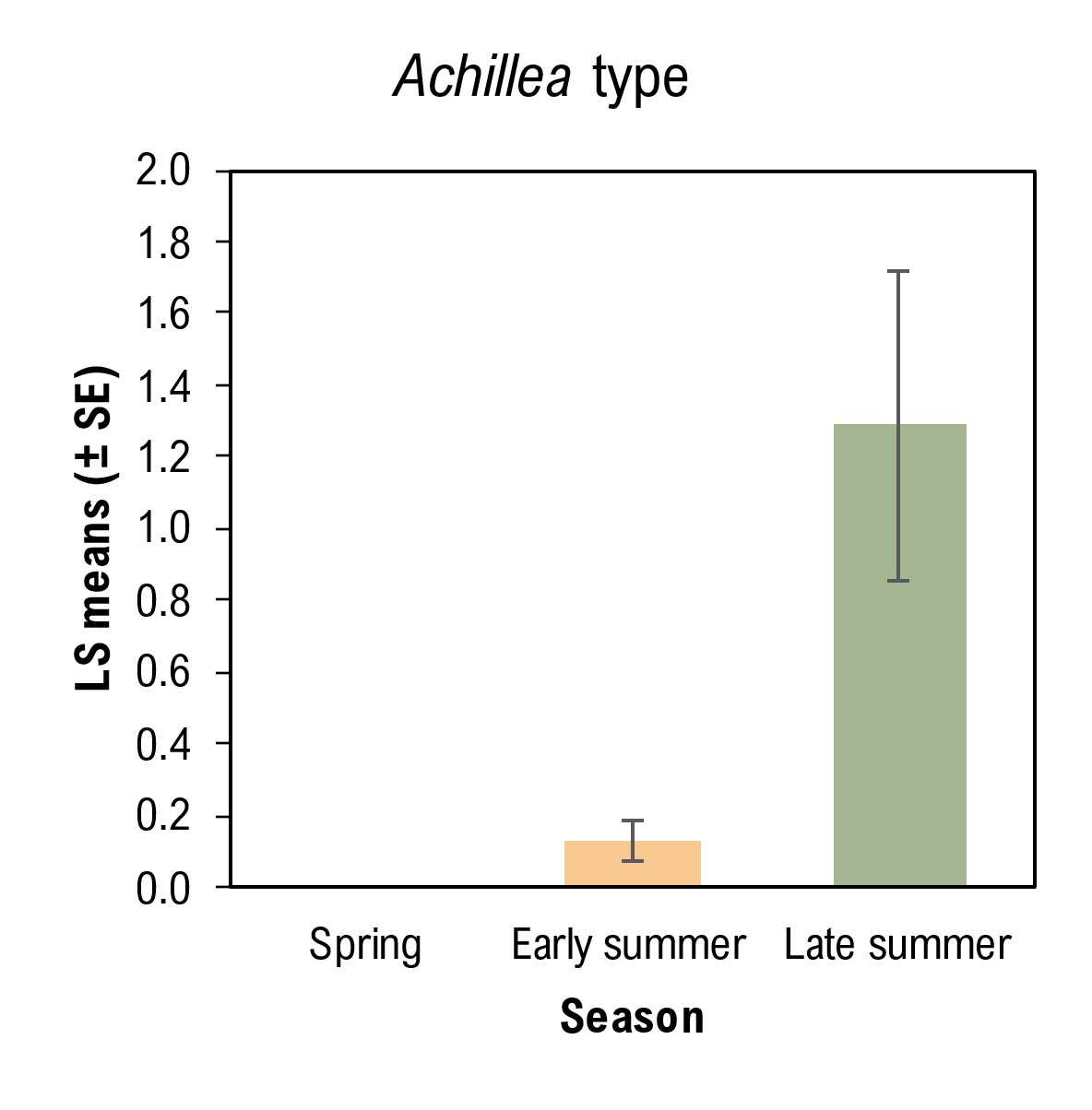


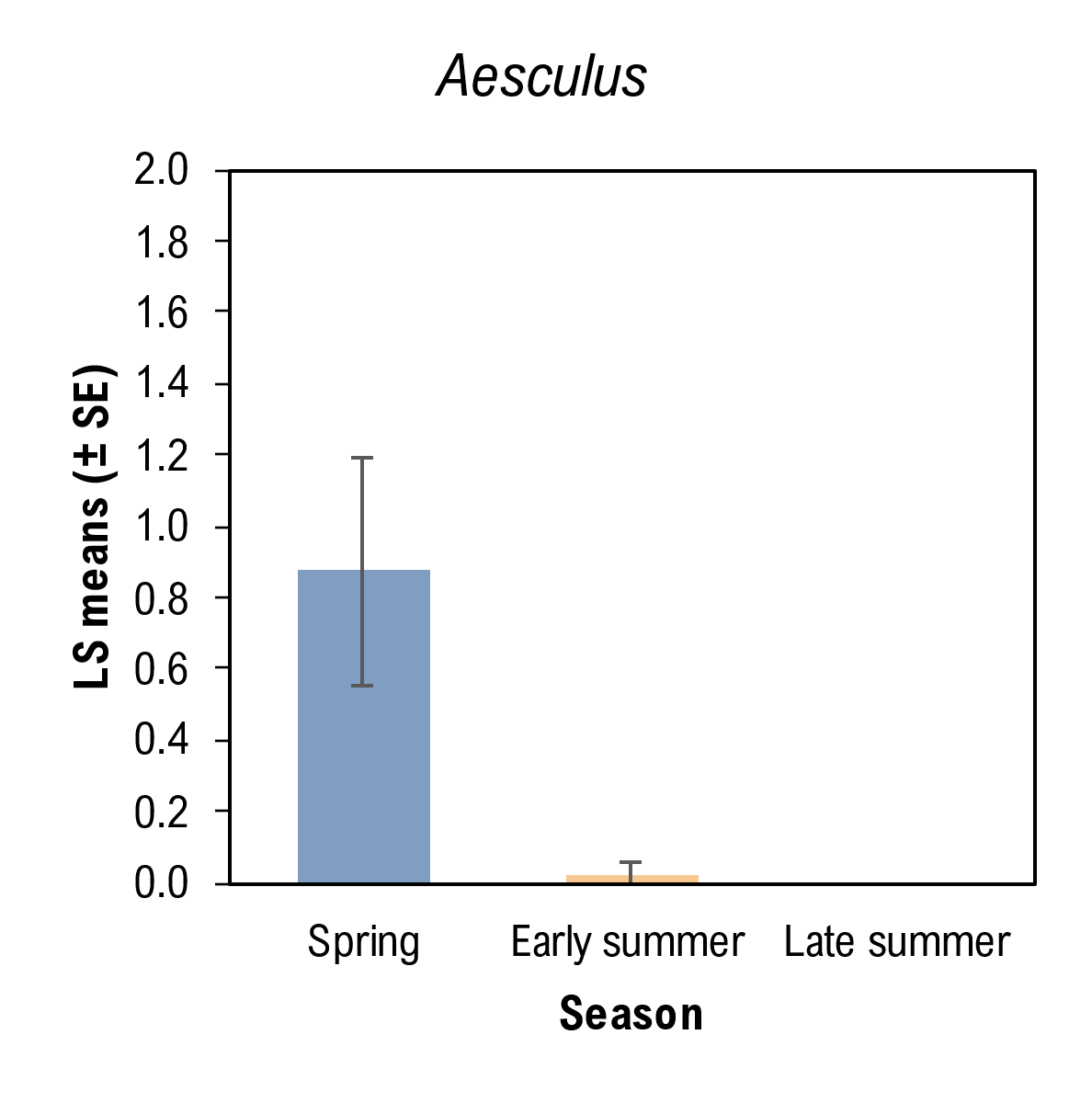

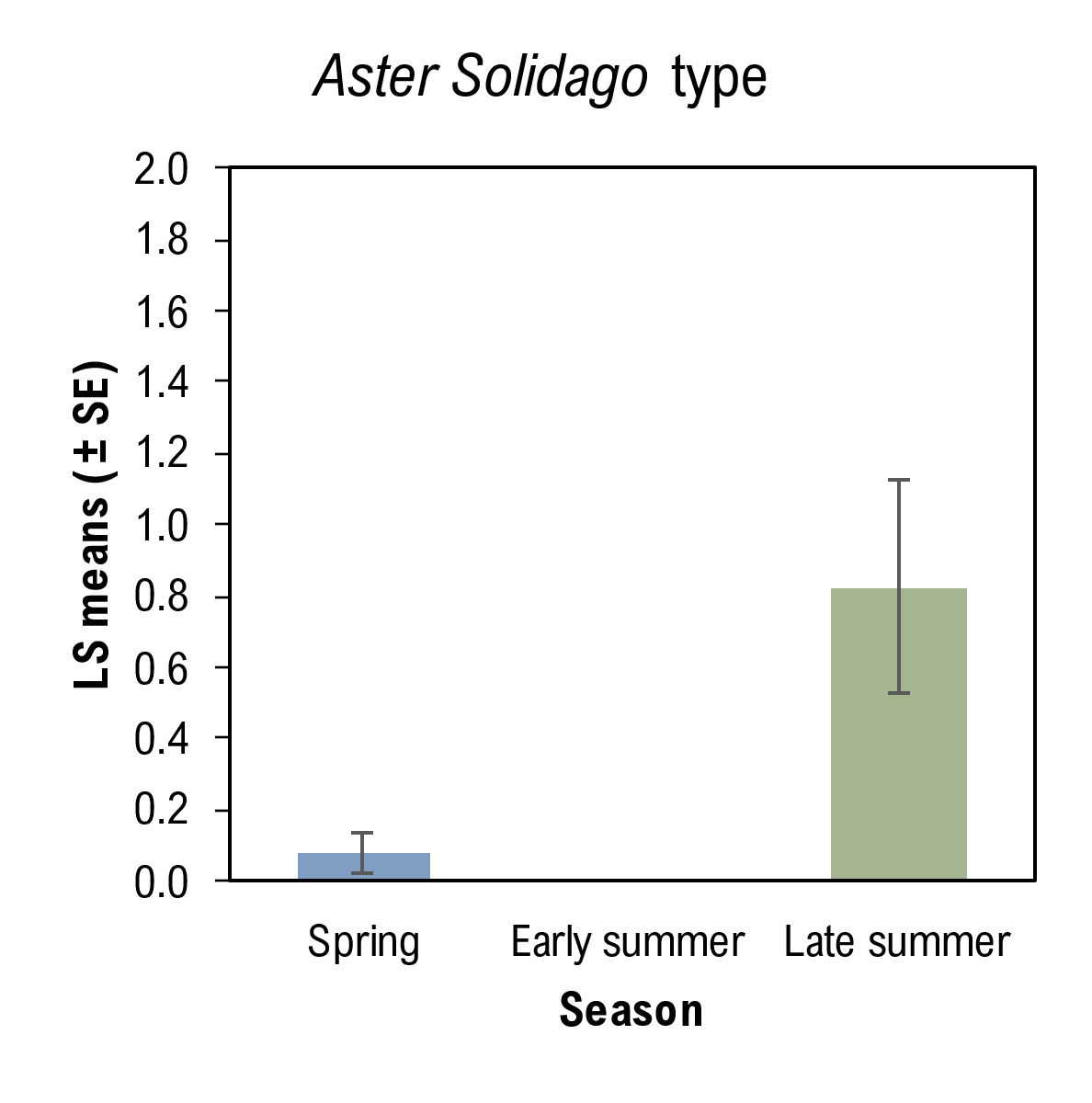


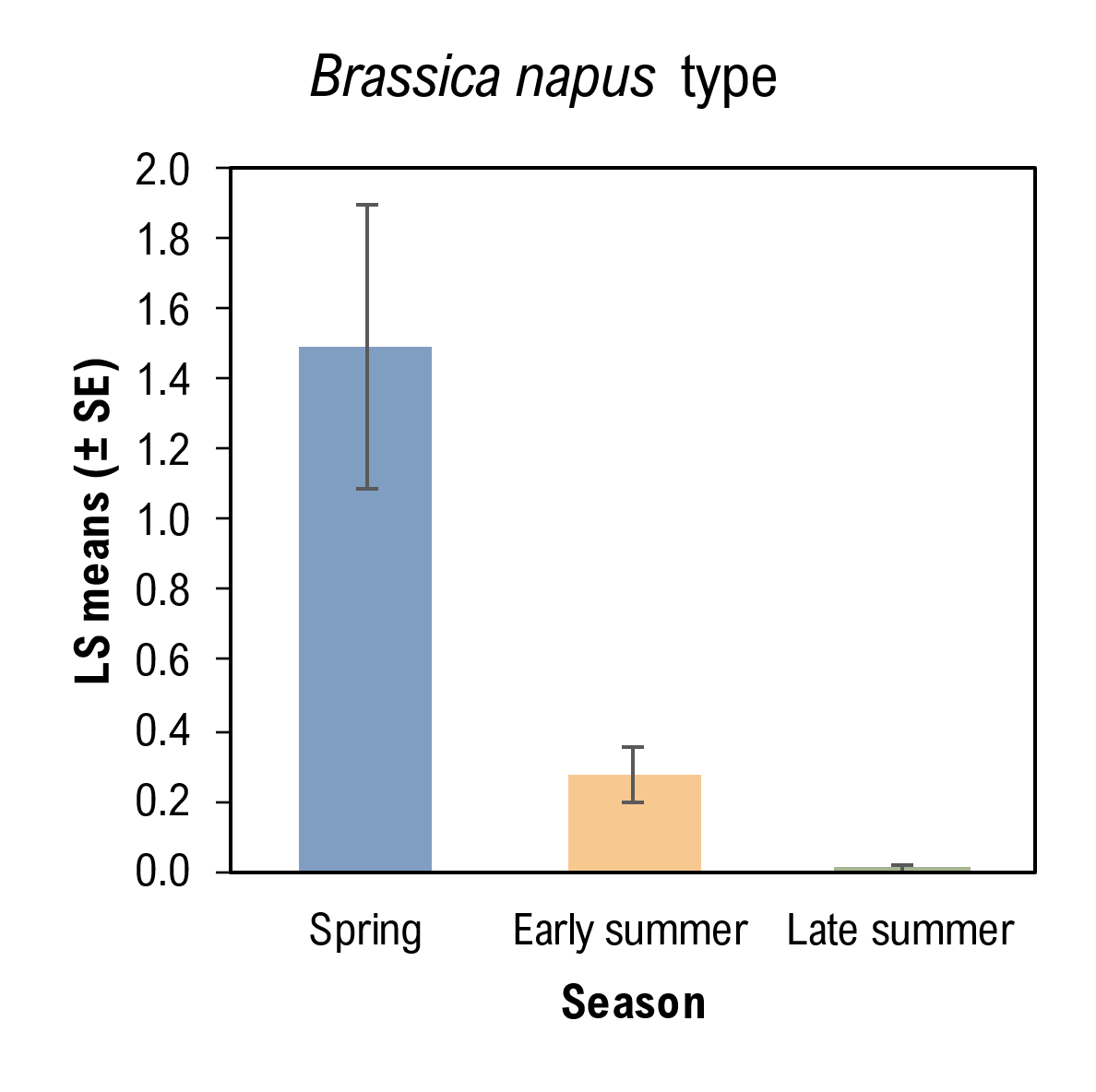

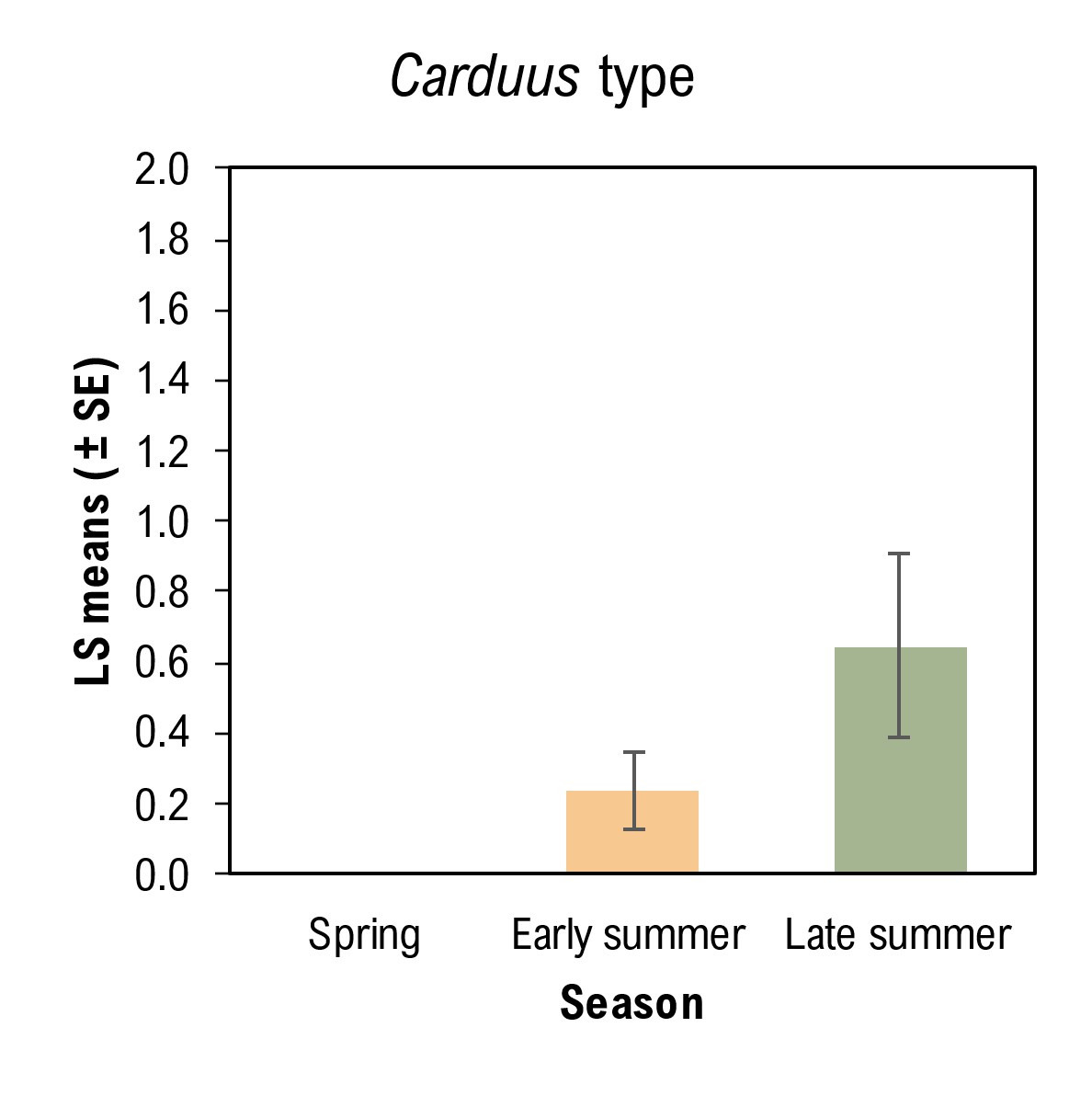


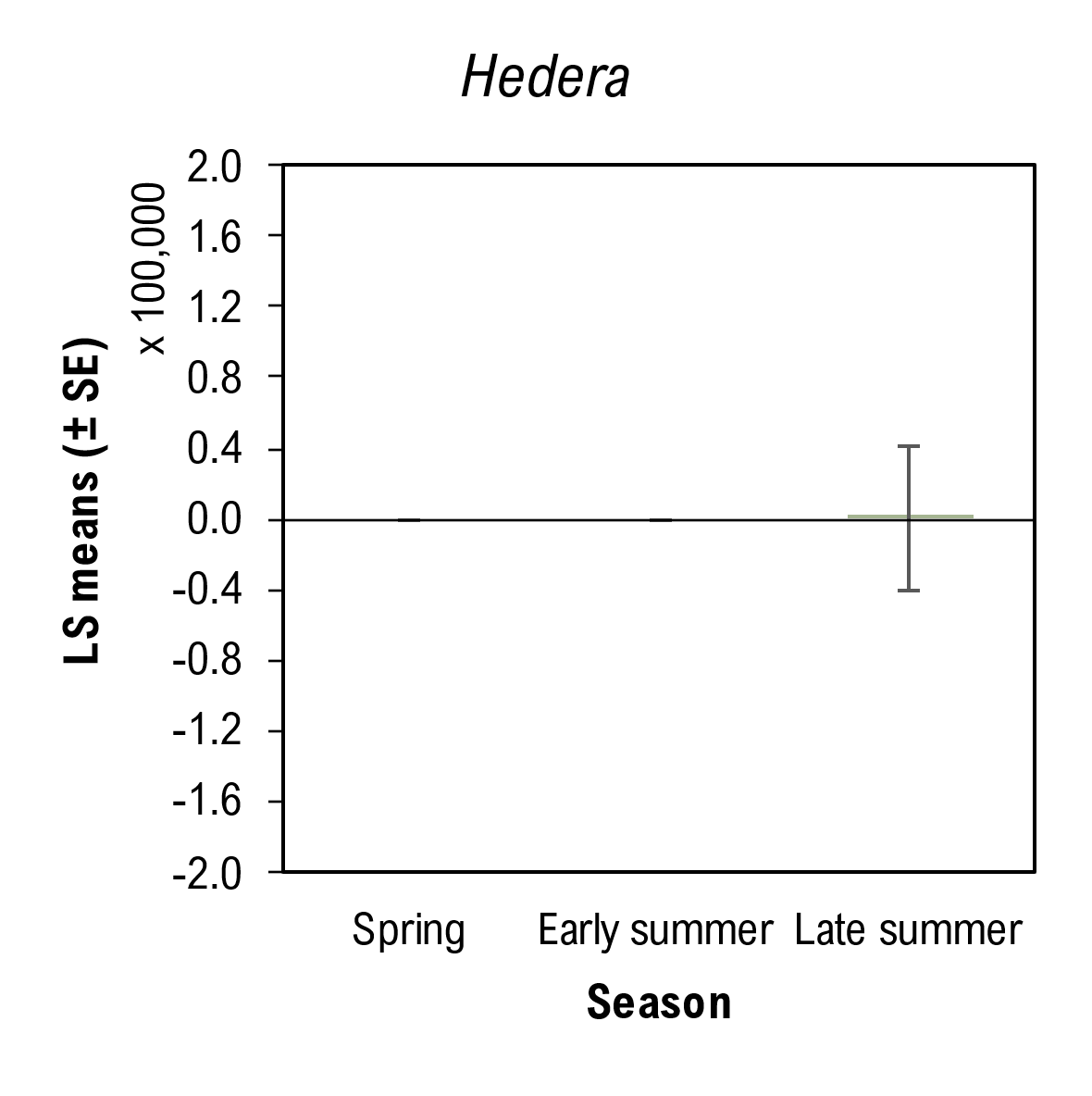

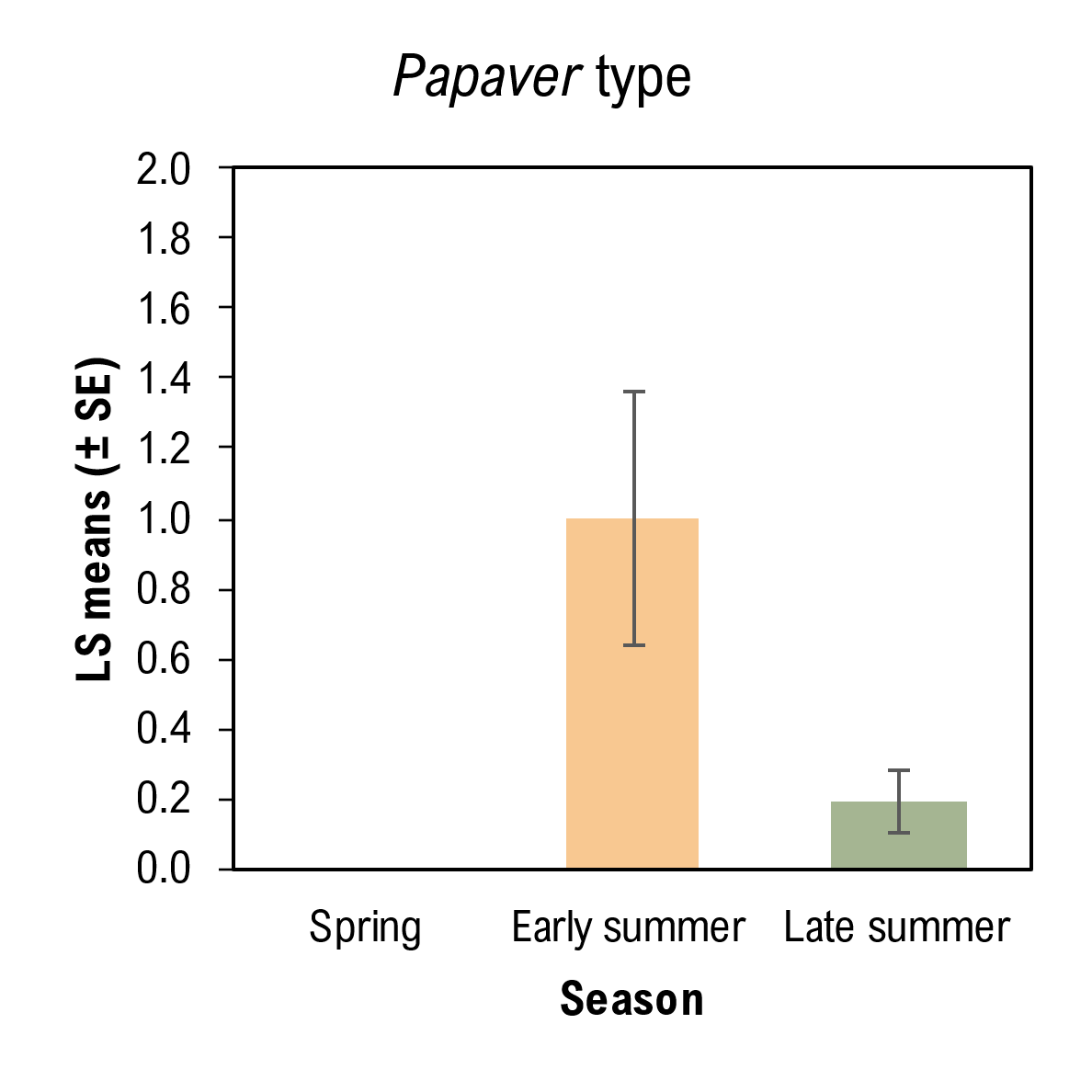


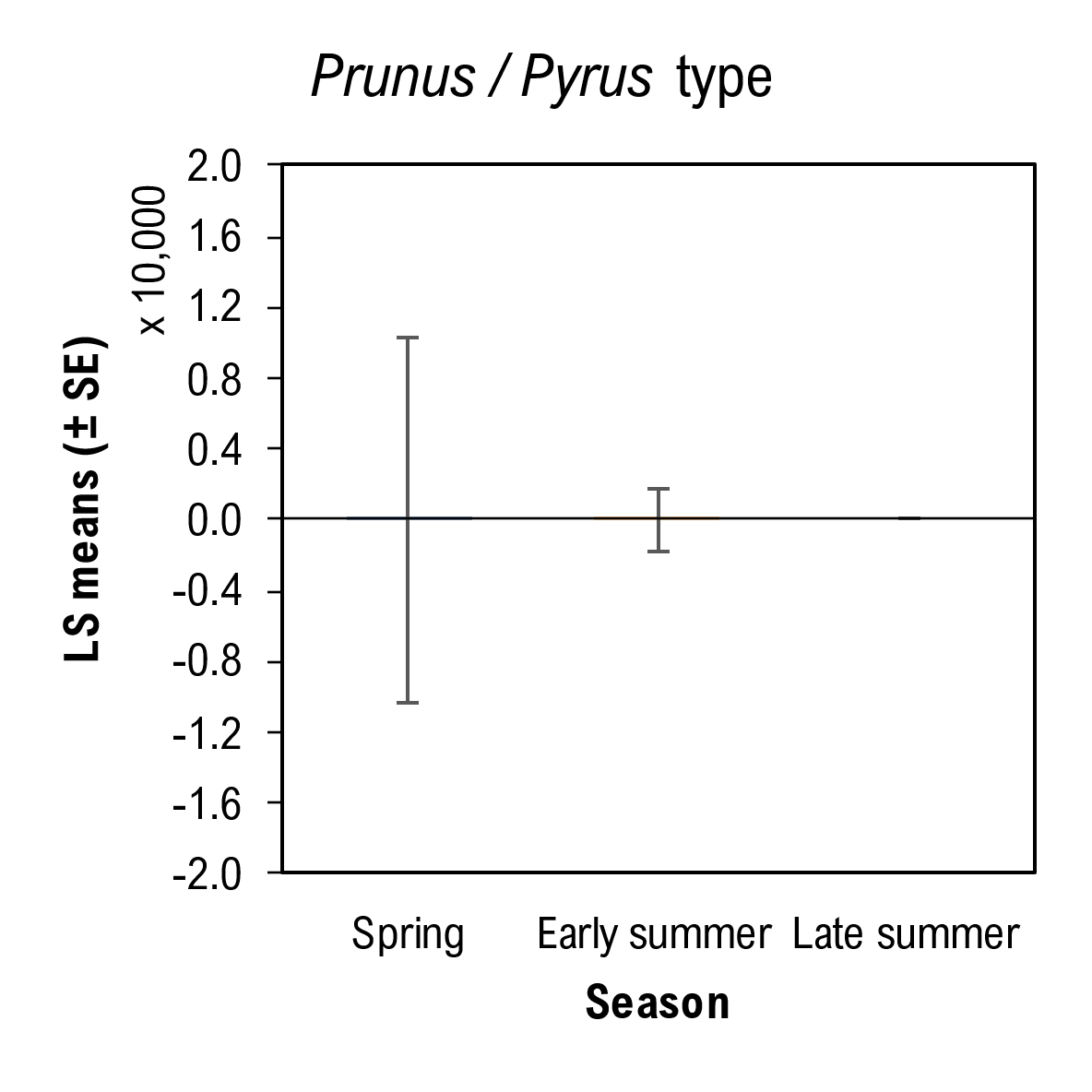

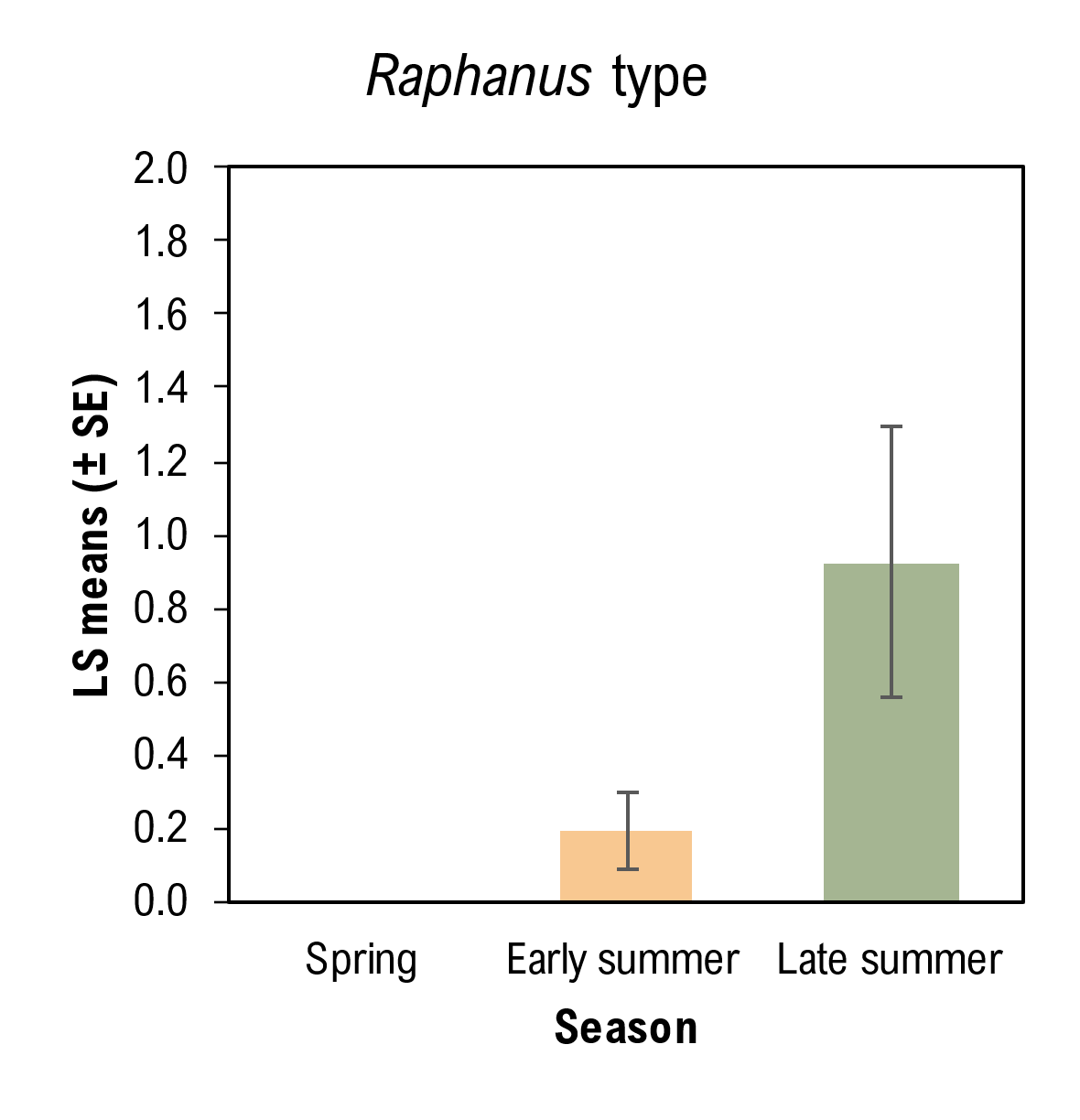


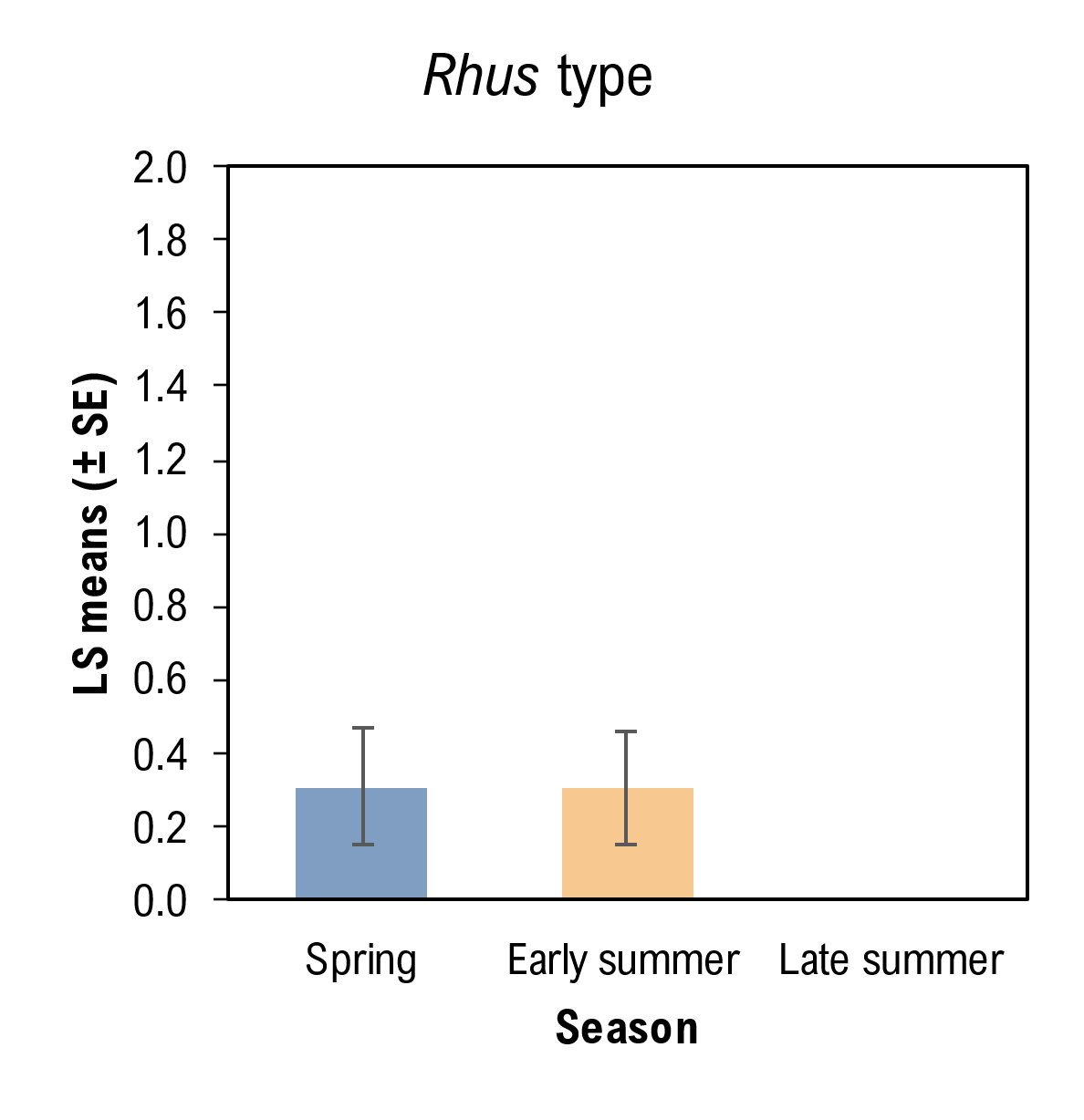

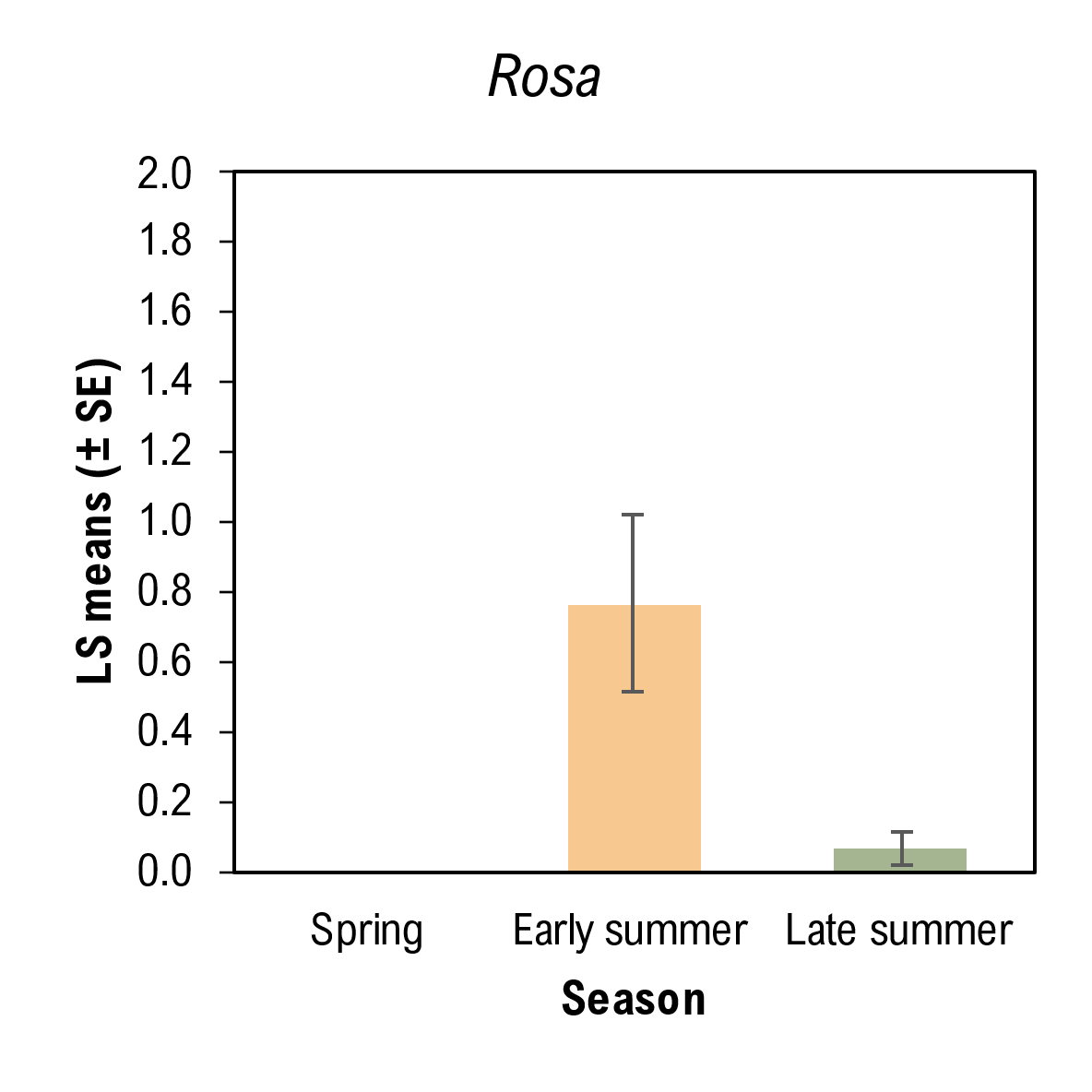


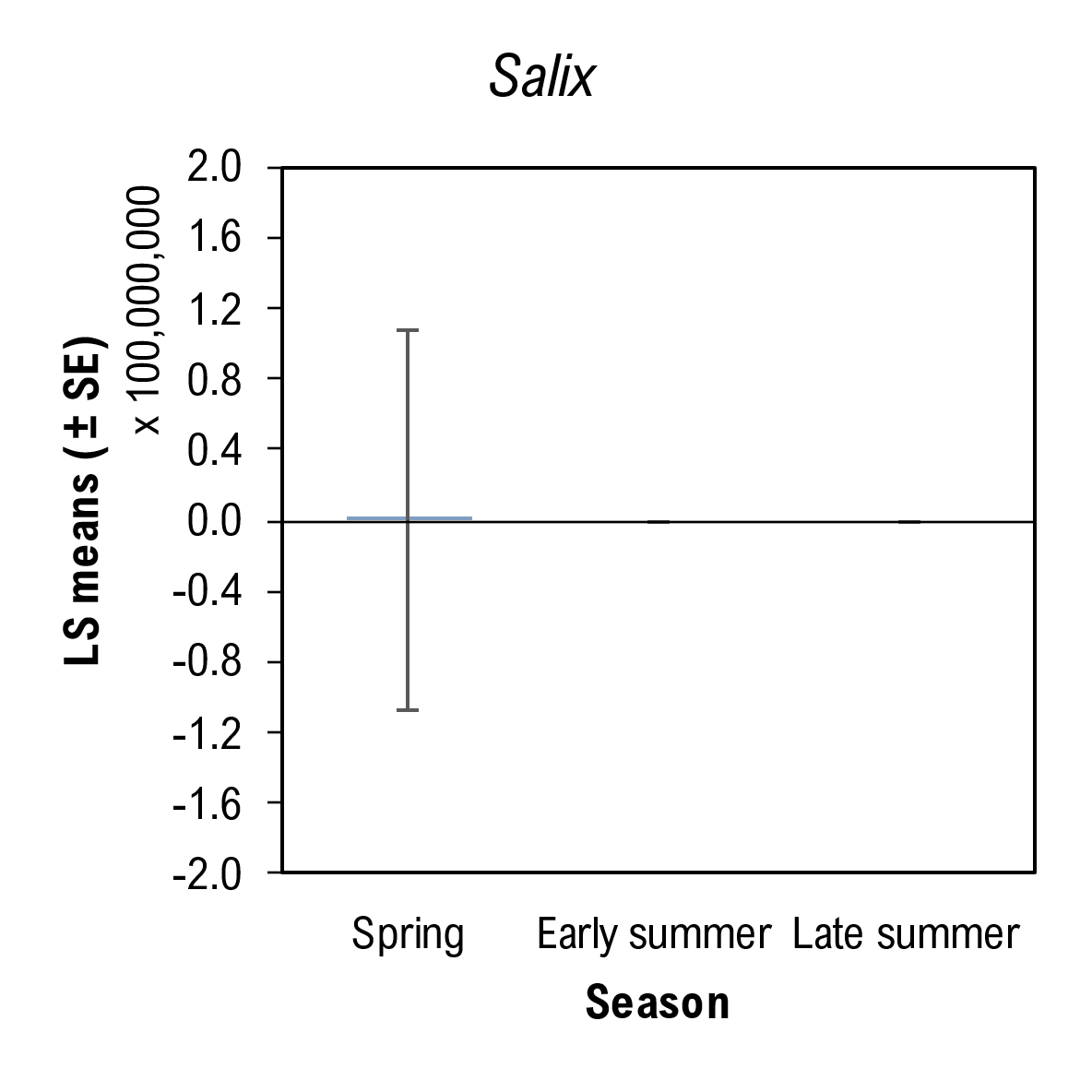

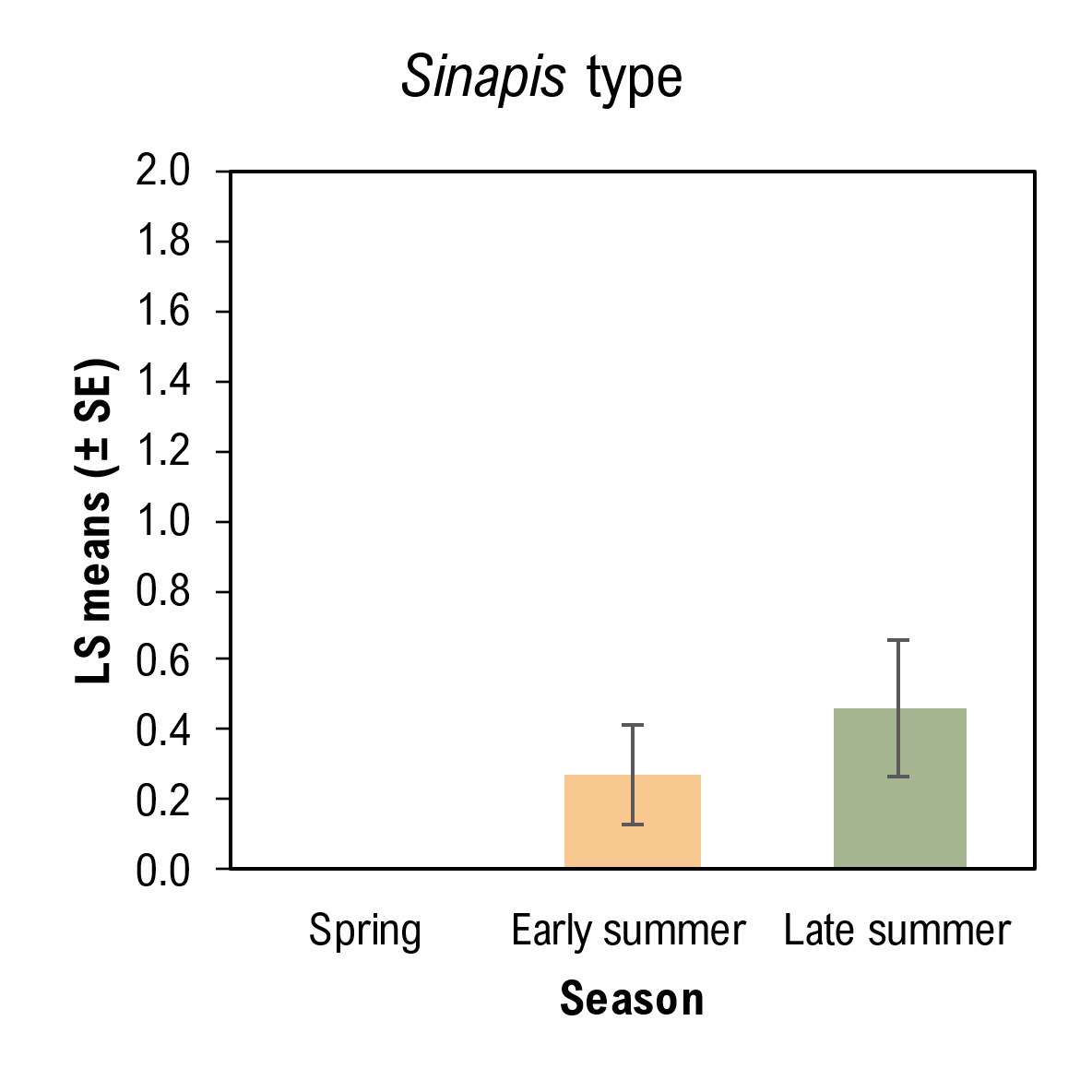


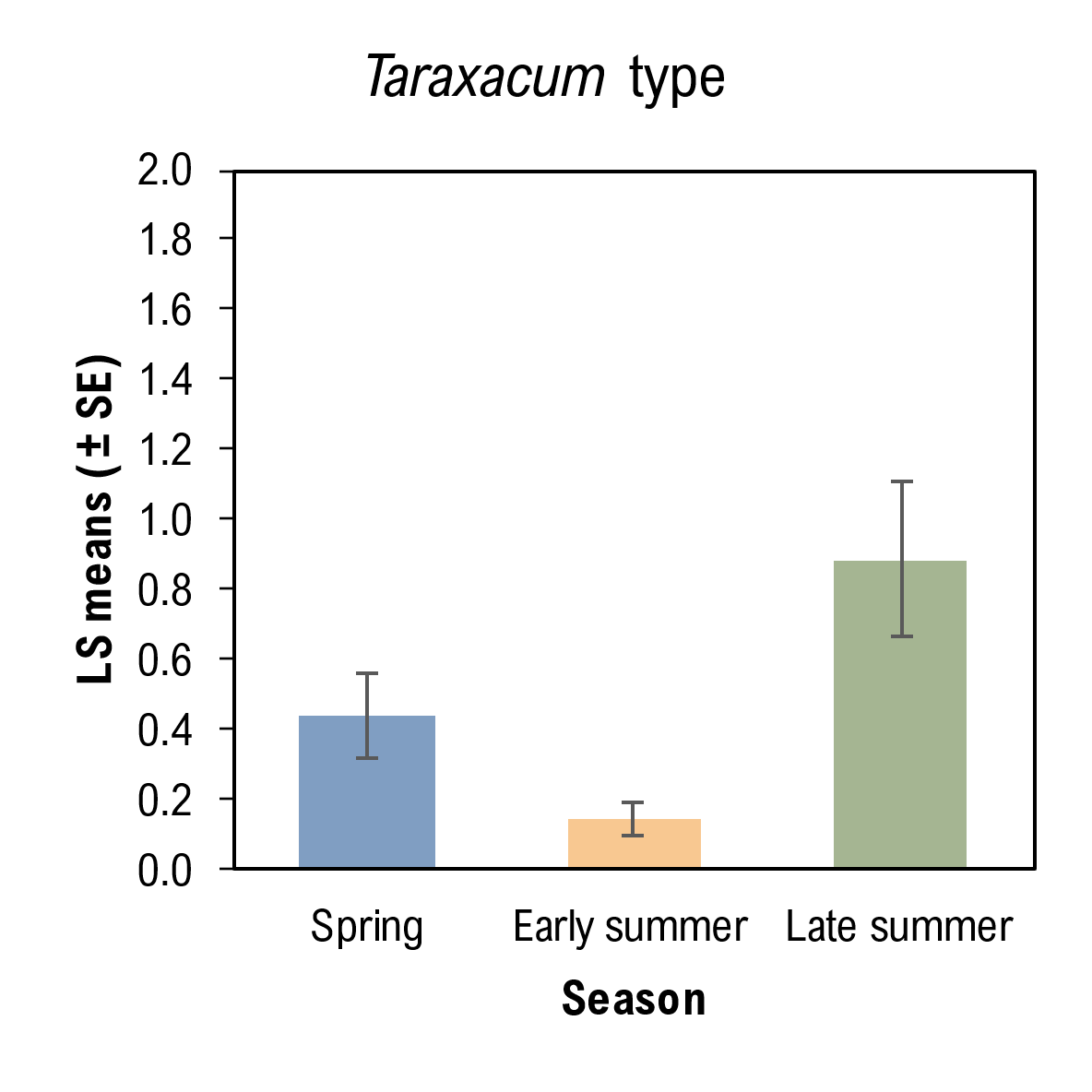

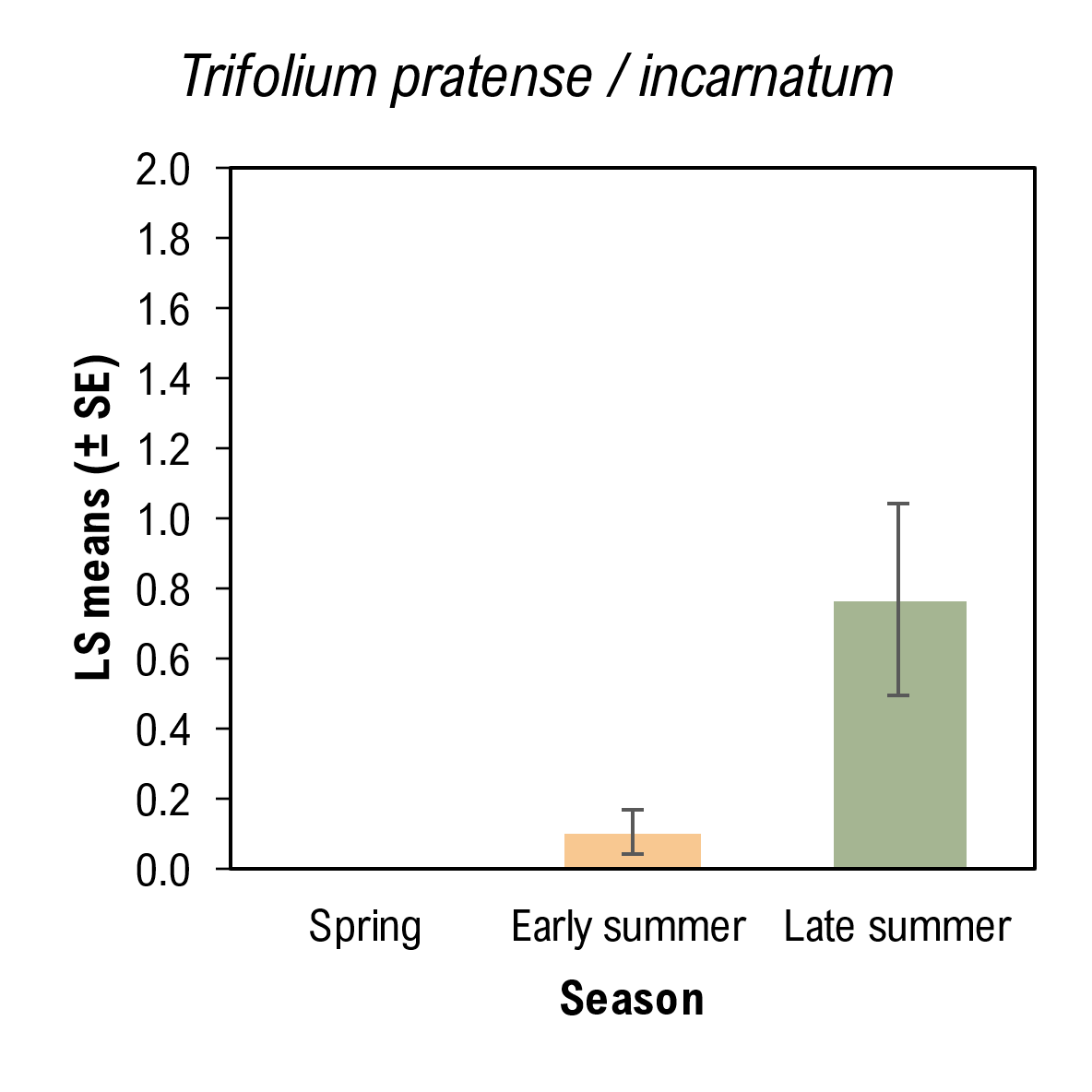


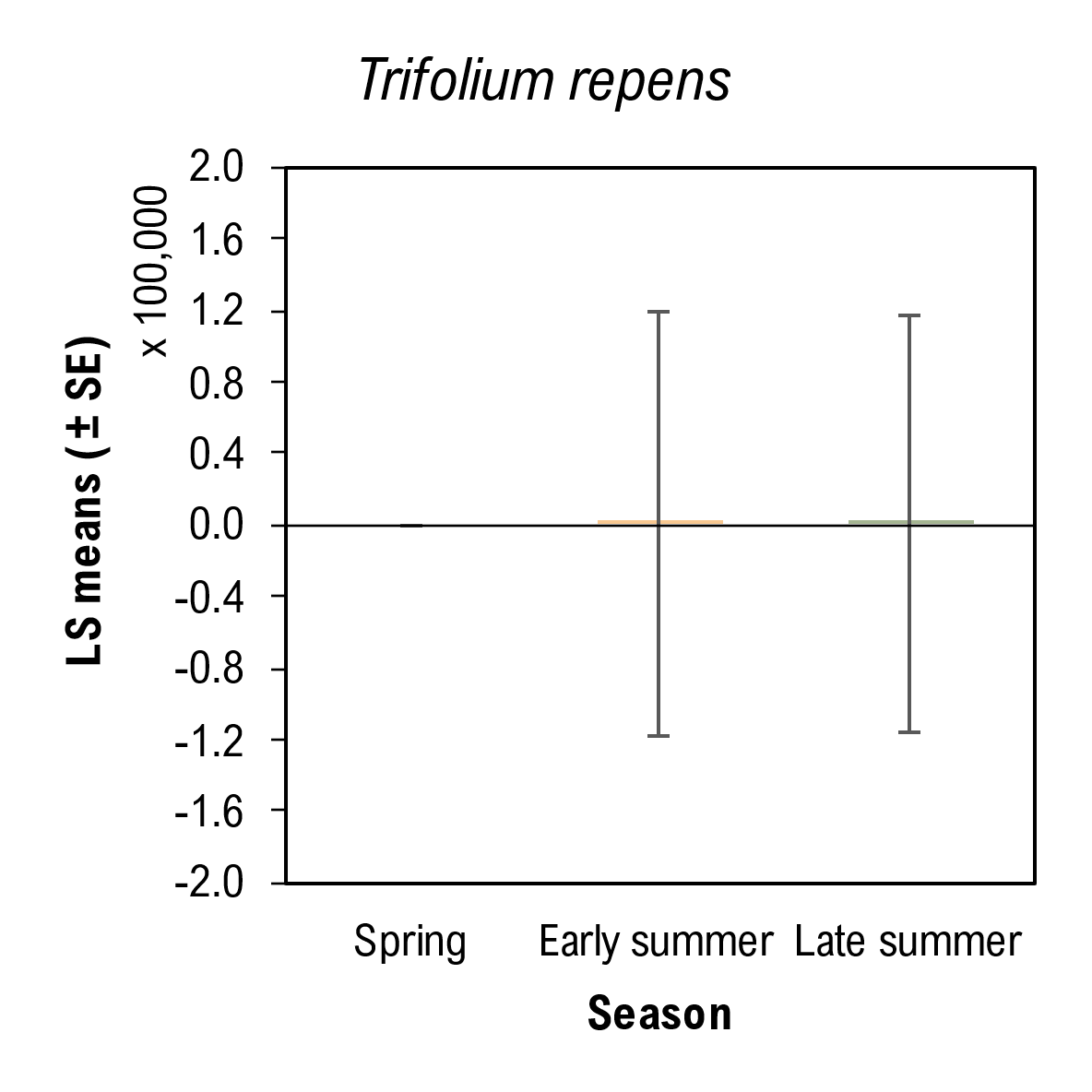


Table S3.1 Least square means differences in pollen diversity for period in Model 1 and Model 2, respectively. df=235.

| **Periods** | |  | **Model 1** | |  | **Model 2** | |
| --- | --- | --- | --- | --- | --- | --- | --- |
| **compared** | |  | **t** | **p** |  | **t** | **p** |
| 1 | 2 |  | -0.05 | 0.957 |  | -0.03 | 0.973 |
| 1 | 3 |  | -0.24 | 0.808 |  | -0.21 | 0.831 |
| 1 | 4 |  | -2.31 | 0.022 |  | -2.24 | 0.026 |
| 1 | 5 |  | -5.92 | 0.000 |  | -5.85 | 0.000 |
| 1 | 6 |  | -5.60 | 0.000 |  | -5.57 | 0.000 |
| 1 | 7 |  | -7.64 | 0.000 |  | -7.61 | 0.000 |
| 1 | 8 |  | -6.69 | 0.000 |  | -6.65 | 0.000 |
| 1 | 9 |  | -4.22 | 0.000 |  | -4.18 | 0.000 |
| 2 | 3 |  | -0.21 | 0.834 |  | -0.20 | 0.842 |
| 2 | 4 |  | -2.53 | 0.012 |  | -2.47 | 0.014 |
| 2 | 5 |  | -6.64 | 0.000 |  | -6.58 | 0.000 |
| 2 | 6 |  | -6.23 | 0.000 |  | -6.22 | 0.000 |
| 2 | 7 |  | -8.60 | 0.000 |  | -8.59 | 0.000 |
| 2 | 8 |  | -7.51 | 0.000 |  | -7.47 | 0.000 |
| 2 | 9 |  | -4.70 | 0.000 |  | -4.68 | 0.000 |
| 3 | 4 |  | -2.41 | 0.017 |  | -2.36 | 0.019 |
| 3 | 5 |  | -6.73 | 0.000 |  | -6.67 | 0.000 |
| 3 | 6 |  | -6.28 | 0.000 |  | -6.28 | 0.000 |
| 3 | 7 |  | -8.76 | 0.000 |  | -8.76 | 0.000 |
| 3 | 8 |  | -7.64 | 0.000 |  | -7.61 | 0.000 |
| 3 | 9 |  | -4.69 | 0.000 |  | -4.68 | 0.000 |
| 4 | 5 |  | -4.44 | 0.000 |  | -4.43 | 0.000 |
| 4 | 6 |  | -4.06 | 0.000 |  | -4.09 | 0.000 |
| 4 | 7 |  | -6.60 | 0.000 |  | -6.63 | 0.000 |
| 4 | 8 |  | -5.40 | 0.000 |  | -5.41 | 0.000 |
| 4 | 9 |  | -2.35 | 0.019 |  | -2.39 | 0.018 |
| 5 | 6 |  | 0.26 | 0.795 |  | 0.22 | 0.827 |
| 5 | 7 |  | -2.20 | 0.029 |  | -2.25 | 0.025 |
| 5 | 8 |  | -0.97 | 0.333 |  | -1.00 | 0.320 |
| 5 | 9 |  | 2.07 | 0.040 |  | 2.02 | 0.045 |
| 6 | 7 |  | -2.37 | 0.018 |  | -2.39 | 0.018 |
| 6 | 8 |  | -1.19 | 0.235 |  | -1.18 | 0.240 |
| 6 | 9 |  | 1.74 | 0.084 |  | 1.73 | 0.085 |
| 7 | 8 |  | 1.24 | 0.215 |  | 1.27 | 0.206 |
| 7 | 9 |  | 4.24 | 0.000 |  | 4.24 | 0.000 |
| 8 | 9 |  | 3.04 | 0.003 |  | 3.01 | 0.003 |

Table S3.2. Effect of landscape variables on abundance (presence of pollen species). Note that all tests were made as univariate tests on log transformed distance and area. If the G matrix was not positive definite we omitted the test from the table.

| **Effect** | **Pollen type analysis** | **df** | **F** | **p** | **Estimate** |
| --- | --- | --- | --- | --- | --- |
| Log Distance_OSR | *Achillea* type | 1. 263 | 2.51 | 0.115 | -0.38 |
|  | *Aster Solidago* type | 1. 263 | 1.45 | 0.230 | 0.48 |
|  | *Carduus* type | 1. 264 | 0.09 | 0.759 | 0.09 |
|  | *Hedera* | 1. 263 | 0.75 | 0.387 | 0.23 |
|  | *Papaver* type | 1. 263 | 8.66 | 0.004 | -0.80 |
|  | *Raphanus* type | 1. 262 | 0.11 | 0.737 | 0.12 |
|  | *Rhus* type | 1. 264 | 5.05 | 0.026 | 0.83 |
|  | *Rosa* | 1. 263 | 1.01 | 0.317 | -0.23 |
|  | *Sinapis* type | 1. 263 | 2.20 | 0.140 | -0.38 |
|  | *Taraxacum* type | 1. 263 | 0.09 | 0.766 | -0.05 |
|  | *Trifolium pratense/incarnatum* | 1. 262 | 3.66 | 0.057 | -0.42 |
|  | *Trifolium repens* | 1. 262 | 1.61 | 0.205 | -0.16 |
| Log Area_green_urban | *Achillea* type | 1. 264 | 0.22 | 0.638 | 0.14 |
|  | *Aster Solidago* type | 1. 264 | 8.17 | 0.005 | 1.48 |
|  | *Carduus* type | 1. 265 | 0.98 | 0.323 | 0.34 |
|  | *Papaver* type | 1. 264 | 1.02 | 0.313 | 0.41 |
|  | *Raphanus* type | 1. 263 | 5.35 | 0.021 | -0.65 |
|  | *Rosa* | 1. 264 | 0.33 | 0.564 | 0.16 |
|  | *Sinapis* type | 1. 264 | 0.04 | 0.832 | 0.07 |
|  | *Taraxacum* type | 1. 264 | 3.21 | 0.074 | -0.28 |
|  | *Trifolium pratense/incarnatum* | 1. 263 | 0.01 | 0.941 | -0.02 |
| Log Area_HNV | *Achillea* type | 1. 264 | 4.88 | 0.028 | 2.05 |
|  | *Aster Solidago* type | 1. 264 | 0.00 | 0.984 | -0.01 |
|  | *Carduus* type | 1. 265 | 0.82 | 0.367 | 0.64 |
|  | *Hedera* | 1. 264 | 2.75 | 0.099 | -0.27 |
|  | *Papaver* type | 1. 264 | 1.23 | 0.268 | 0.97 |
|  | *Raphanus* type | 1. 263 | 0.07 | 0.785 | 0.11 |
|  | *Rhus* type | 1. 265 | 3.03 | 0.083 | -0.36 |
|  | *Rosa* | 1. 264 | 0.23 | 0.632 | -0.11 |
|  | *Sinapis* type | 1. 264 | 0.23 | 0.634 | 0.22 |
|  | *Taraxacum* type | 1. 264 | 5.43 | 0.021 | 0.89 |
|  | *Trifolium pratense/incarnatum* | 1. 263 | 0.02 | 0.882 | 0.04 |
|  | *Trifolium repens* | 1. 263 | 4.68 | 0.031 | 0.40 |
| Log Area_pollencrop_medium | *Achillea* type | 1. 263 | 0.10 | 0.753 | 0.09 |
|  | *Aster Solidago* type | 1. 263 | 0.04 | 0.845 | -0.06 |
|  | *Carduus* type | 1. 264 | 0.23 | 0.628 | 0.15 |
|  | *Papaver* type | 1. 263 | 0.02 | 0.891 | 0.04 |
|  | *Raphanus* type | 1. 262 | 0.07 | 0.796 | 0.09 |
|  | *Rhus* type | 1. 264 | 0.07 | 0.797 | 0.08 |
|  | *Rosa* | 1. 263 | 0.91 | 0.340 | -0.24 |
|  | *Sinapis* type | 1. 263 | 1.53 | 0.217 | -0.39 |
|  | *Taraxacum* type | 1. 263 | 0.48 | 0.490 | 0.12 |
|  | *Trifolium pratense/incarnatum* | 1. 262 | 1.25 | 0.265 | 0.33 |
|  | *Trifolium repens* | 1. 262 | 1.68 | 0.196 | 0.18 |
